# Laws of concatenated perception: Vision goes for novelty, decisions for perseverance

**DOI:** 10.1371/journal.pbio.3000144

**Published:** 2019-03-05

**Authors:** David Pascucci, Giovanni Mancuso, Elisa Santandrea, Chiara Della Libera, Gijs Plomp, Leonardo Chelazzi

**Affiliations:** 1 Department of Neuroscience, Biomedicine and Movement Sciences, University of Verona, Verona, Italy; 2 Department of Psychology, University of Fribourg, Fribourg, Switzerland; 3 National Institute of Neuroscience, Verona, Italy; Vanderbilt University, UNITED STATES

## Abstract

Every instant of perception depends on a cascade of brain processes calibrated to the history of sensory and decisional events. In the present work, we show that human visual perception is constantly shaped by two contrasting forces exerted by sensory adaptation and past decisions. In a series of experiments, we used multilevel modeling and cross-validation approaches to investigate the impact of previous stimuli and decisions on behavioral reports during adjustment and forced-choice tasks. Our results revealed that each perceptual report is permeated by opposite biases from a hierarchy of serially dependent processes: Low-level adaptation repels perception away from previous stimuli, whereas decisional traces attract perceptual reports toward the recent past. In this hierarchy of serial dependence, “continuity fields” arise from the inertia of decisional templates and not from low-level sensory processes. This finding is consistent with a Two-process model of serial dependence in which the persistence of readout weights in a decision unit compensates for sensory adaptation, leading to attractive biases in sequential perception. We propose a unified account of serial dependence in which functionally distinct mechanisms, operating at different stages, promote the differentiation and integration of visual information over time.

## Abstract 2

A combination of psychophysics and computational modeling reveals the independent effects of past stimuli and decisions on single perceptual reports, with functionally distinct mechanisms promoting the differentiation and integration of visual information over time.

## Author summary

What occurred a moment ago has a strong impact on how we perceive the present: previous sensory events, decisions, and actions all leave traces that can affect our current perception. In this study, we demonstrate that human perception is constantly shaped by two contrasting forces arising from the history of sensory and decisional events. On the one hand, repulsive forces push perception away from recent stimuli. On the other, attractive forces dominate human perception during sequences of perceptual decisions, biasing the present sensory input to appear more similar to the past than it actually is. We have formalized these findings in a computational, physiologically informed model that takes into account the hierarchical structure of perceptual processing in the human brain, proposing a unifying view of history biases in perception.

## Introduction

From moment to moment, human perception is not a high-fidelity replica of sensory input but relies heavily on past experience and short-term dependencies: what happened a moment ago has a strong impact on how we perceive the present.

Vision, for example, is inherently contaminated by the immediate past: low-level visual features such as brightness, color, or motion are all perceived depending on their temporal context [1–3], and when the stimulation history changes, identical stimuli may well lead to dissimilar percepts [1,4,5]. Such short-term dependencies are not peculiar to visual perception but permeate a wide range of cognitive processes, including attention [6–9], decision-making [10–12], memory [13,14], confidence in performance [15], and motor behavior [16]. This implies that, at multiple stages, our cognitive system is anchored and calibrated to the recent history of sensory and decisional processes.

From a phenomenological perspective, short-term dependencies may carry opposite effects on perception: negative (repulsive) effects are observed when perception is repelled away from previous stimuli [1,4,17,18]; positive (attractive) effects, instead, arise when consecutive stimuli appear more similar than they really are, and thus past representations persist and “attract” current perceptions.

Attractive and repulsive biases evoked by the history of stimuli, behavioral responses, and memory content have been recognized ever since the dawn of psychophysical and psychological research [11,19–25], but the interest has now regrown considerably after the recent work of Fischer and Whitney [26]. In their collection of experiments, human participants were asked to report the orientation of visual stimuli, trial after trial, using adjustment responses. The authors found that adjustment errors were systematically attracted toward the orientation of stimuli seen one or a few trials before [26]. This led to the hypothesis of a specific neural mechanism, termed the “continuity field,” that would promote positive serial dependence at the earliest stages of sensory coding [27]. Continuity fields have been reported afterwards in a variety of perceptual tasks [28–33] and for features encoded at multiple stages along the visual hierarchy, including for basic stimulus features, such as orientation [26], spatial position [34], and numerosity [28], but also for more complex stimuli, such as faces [35], emotion [36], and facial attractiveness [37]. Such ubiquity of positive biases has led some authors to hypothesize that serial dependence is an intrinsic mechanism through which our visual system exploits temporal correlations and contextual redundancies by merging similar stimuli, slightly changing over time, into a coherent and “continuous” perceptual field [26,27].

Although the idea of a continuity field fits neatly with recent findings, it stands in sharp contrast with other, widely documented forms of negative dependence. Examples of negative serial dependence are well-known phenomena of visual adaptation and aftereffects [18]. In visual aftereffects, previous exposure to persistent features, such as orientation [4], color [18] or motion [1], causes subsequent stimuli to appear as shifted away from the exposed feature: a stationary object will seem to be moving downward after exposure to upward motion; a vertical grating will look as if it is tilted clockwise after exposure to counterclockwise orientations. These negative biases go plainly in the opposite direction to that predicted by the continuity field, and thus the question arises as to how these two contrasting forces, one attractive and the other repulsive, may coexist in perceptual processing [38].

Although there is a wealth of evidence linking negative aftereffects to transitory decreases in the responsivity of relevant populations of sensory neurons [39–42], less is known about the mechanisms regulating positive serial dependence: is there a shared mechanism responsible for both types of dependencies, or are the two phenomena related to distinct and independent stages of visual processing [30]?

According to the original account of serial dependence [26], continuity fields emerge at the very early stages of processing [29,43], likely from the persistence of past activity patterns in sensory areas [26,44]. This view, and the related computational framework [26], suggested a physiological process that closely resembled neuronal adaptation but with an opposite and conceivably distinct outcome: sensory neurons encoding previous stimuli increase their responsivity to future events, thus biasing perceptual processing toward the recent past [26,29]. This entails the coexistence of two additive [29,45] or opposite [30] processes—adaptation and serial dependence—that compete in pulling the appearance of stimuli toward or away from the past [26,29,46]. To explain what factors may determine the prevalence of one or the other, a major role has been assigned to the nature of sensory input: strong and persistent stimuli cause neuronal adaptation and the resulting repulsion [2]; weak, uncertain, and briefly presented stimuli promote positive serial dependence [29]. Since the functional role of these opposite biases appears closely related to the integration (positive serial dependence) and segregation (adaptation) of visual events over time, it has been also suggested that their relative prevalence might depend on the stability or inconstancy of stimuli in the natural environment, with attractive biases for stable attributes (e.g., the identity or gender of a face) and repulsive biases for changeable features (e.g., facial expressions) [32], hence conferring a major role to prior knowledge and environmental statistics in determining the direction of biases in a sequence of perceptual events [38,47].

The issue has been recently raised, however, about the stage at which positive serial dependence originates. Several studies, in fact, have presented evidence for opposite effects of the recent past at different stages of processing [30,48]: repulsive biases, akin to negative aftereffects [2,18], at the early stages of perception and attractive biases emerging later and increasing in magnitude as the postperceptual time before a behavioral response increased [30,48]. In light of these findings, an alternative account has been proposed that advocates the coexistence of two types of serial dependence in perceptual judgments: negative dependence evoked by short-term adaptation and positive dependence caused by postperceptual biases in working memory [30,48]. This alternative account denied the low-level and “perceptual” nature of serial dependence, favoring a postperceptual explanation in which previous sensory events do not directly attract the appearance of stimuli [30,48].

Although positive serial dependence at the early stages of perception has been confirmed in more recent studies [29,43,49], the overall scenario remains controversial. First, opposite history biases have been reported under very similar circumstances and using virtually identical stimuli [26,29,30], which makes it unlikely that the only decisive factor is the kind of sensory input. Second, although positive serial dependence has been observed in response to weak and noisy sensory signals, it nevertheless requires attentional resources (but see [50]), whereas repulsive biases come about after nonattended stimuli [26]. Moreover, the increase in neuronal responsivity, which putatively underlies positive serial dependence [26], is a well-known mechanism of gain modulation triggered by feature-based attention [51], but to the best of our knowledge, there is no physiological evidence of the persistence of such increase for several seconds after stimulus offset. Finally, although the two accounts reviewed above make dissociable predictions about the locus of positive serial dependence and its effect on stimulus appearance, other interpretations are equally possible [30,47,52], and a theoretical framework to resolve this puzzling interplay between adaptation and positive dependencies is required.

In the present work, we propose and test a new account based on the notion of decisional inertia [53] and inspired by the hierarchy of recurrent neuronal processes that serve the needs of perceptual decision-making [54–58]. We hypothesized that positive serial dependence originates from the persistence of internal templates [59,60] formed during the weighting of past sensory evidence into perceptual decisions, which then become attractor points for future perceptual representations. With the term “template,” here we refer to the profile with which sensory information is accumulated from a specific region in the stimulus space (e.g., a visual orientation) and integrated into internal representations and decisional outcomes [53–58,61], a process that may involve the contribution of higher-level processing stages (e.g., decisional) [30,57,58,61]. For clarity, we will refer to the persistence of such internal templates over time with the term “decisional inertia” [53]. The novelty of our proposal lies in the fact that positive serial dependence by decisional inertia does not deny the impact of previous events on stimulus appearance (i.e., at the “time of perception” [43,48]); neither does it confine attractive biases to the domain of postperceptual processes. In fact, we reasoned that decisional inertia might alter visual perception independently of the content in working memory but exclusively as a consequence of chains of task-relevant internal representations—decisional events. In line with this hypothesis, we provide compelling evidence for the existence of two separate and functionally distinct stages of visual serial dependence [30,56,62], determined by the independent history of sensory and decisional processes.

In a series of experiments, we measured the ability of human participants to reproduce the orientation of consecutive stimuli, and with the aid of multilevel models and cross-validation techniques, we demonstrate that past perceptual decisions, rather than physical stimuli, represent the effective sources of attractive history biases. We found that stimulus-driven serial dependence disappeared when participants were explicitly instructed to withhold their response, whereas repulsive biases emerged under this condition. This result was corroborated by the striking finding that, after streaks of nonreported orientations, positive serial dependence was still dictated by the most recent perceptual decision. This dependency on previous decisions was not the mere consequence of hysteresis in motor responses, as it was strongly modulated by stimulus contrast and had a direct impact on perceptual sensitivity. We further show that the effect of decisional traces generalizes to other tasks and stimuli and is independent of the content in visual working memory.

Consistent with our results, we propose a hierarchical model of short-term dependencies in which repulsive biases emerge as a result of low-level adaptation and positive serial dependence arises from the reverberation of internal templates reflecting the reading-out of sensory signals from higher-level decision integrators. Whereas rapid negative aftereffects in response to task-irrelevant stimuli may promote exquisite perceptual sensitivity to relevant environmental changes, decisional traces may reflect the reinstatement of the most informative sensory channels in the recent past, thus promoting perceptual continuity and increasing the efficiency of decisional templates over the long term, as reported in perceptual learning (PL) studies [54].

Our work provides a new perspective on the history of sensory and decisional processes by reconciling opposite forms of short-term dependencies into a unified account of concatenated perception and decision-making.

## Results

### Experiment 1: Response versus stimulus

As a primary goal, we addressed whether serial dependence in visual perception depends more on past physical stimuli or on the response decision in the previous trial. Recent work has shown that past stimuli systematically bias the decision about the present sensory input [26,30,63]. However, the history of decisions may itself represent a source of perceptual bias that combines with or even outweighs the impact of previous stimuli [30,53]. To test this possibility, in the present experiment (Experiment 1) we compared the ability of sensory stimuli and perceptual decisions to predict future errors in an orientation adjustment task [26].

**Fig 1:**
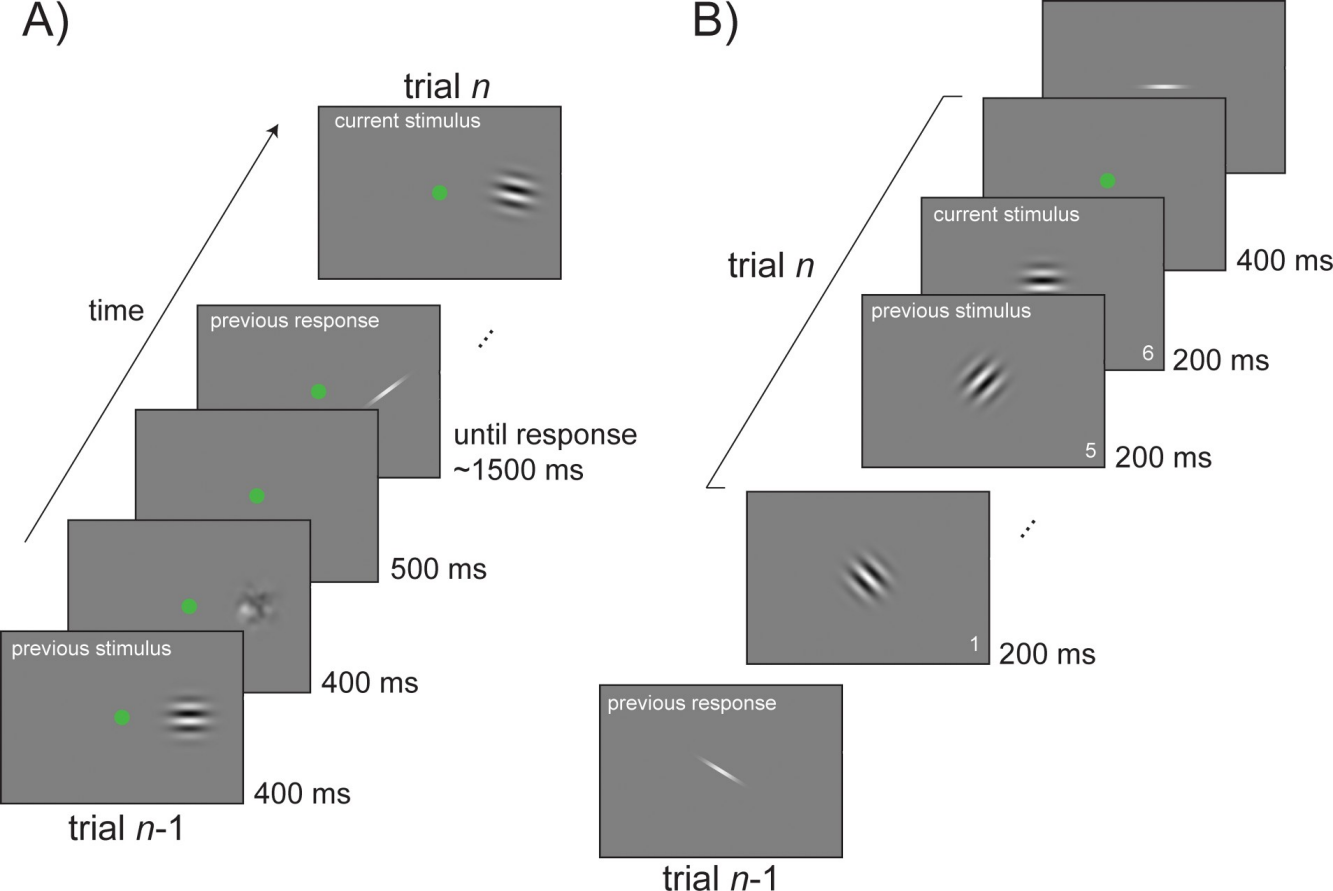
Sequence of events for orientation adjustment tasks. (A) An example of a standard trial in Experiments 1, 2, 3, and 6. Participants viewed a single Gabor in each trial and reported its orientation by adjusting a response bar. In Experiment 2, stimuli were presented at the fovea. In Experiments 1, 3, and 6, stimuli were presented to either the left or right on separate runs. (B) A standard sequence of stimuli in Experiment 5. In this experiment, responses were interleaved with sequences of new stimuli. In a single trial, participants had to pay attention to an entire sequence of stimuli and to report the orientation of the last one. The sequence ended after the sixth stimulus in 80% of the trials and stopped earlier (at a random position in time) in the remaining 20% (catch trials).

**Fig 2:**
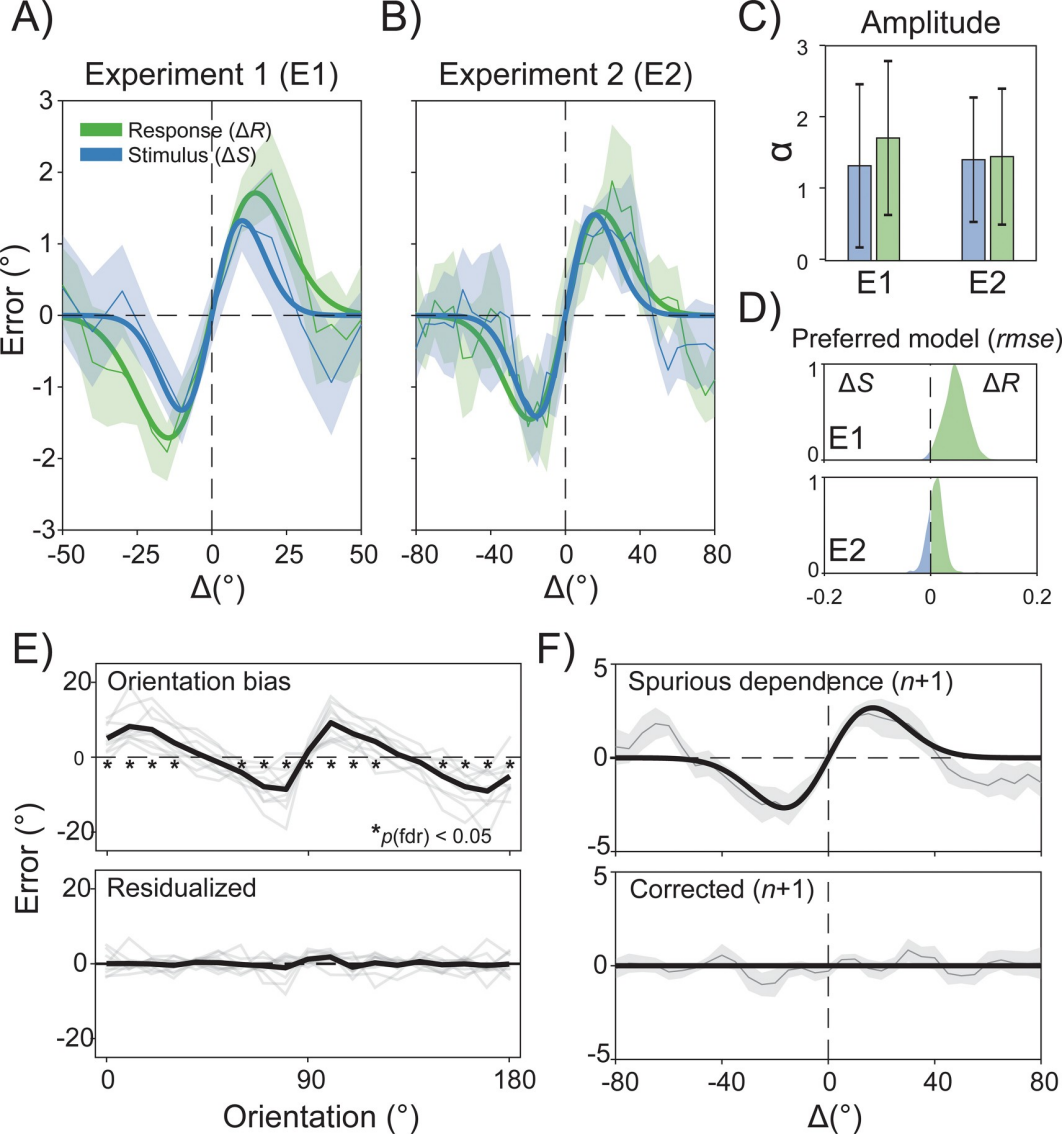
Past responses predict perceptual errors better than previous stimuli. (A–B) Average errors and best-fitting DoG curves from multilevel modeling as a function of previous stimuli (Δ*S*, blue curve and blue shaded bars) or responses (Δ*R*, green curve and green shaded bars) in Experiments 1 (A) and 2 (B). (C) The magnitude of serial dependence (α) is reported for both Δ*S* and Δ*R* (bars on the left side for Experiment 1, right side for Experiment 2). (D) Across experiments, the Δ*R* model showed a remarkably higher ability to predict new data, as revealed by the normalized distributions of rmse (Δ*S* > Δ*R*) obtained through cross-validation. (E) Data were analyzed after residualizing individual adjustment responses from a significant orientation-dependent bias (upper plot; the black line is the average across participants; gray lines are individual average errors; asterisks indicate significance after correction for fdr). The orientation bias confound was successfully removed after fitting a sinusoidal function to the data (lower plot). (F) Using the data of Experiment 2 as a representative case, we show the amount of spurious serial dependence induced by the orientation bias dependence when Δ*R* is computed for one trial in the future (upper plot). After residualization, artefactual serial dependence was successfully removed (lower plot). Note that for this and all subsequent experiments with a discrete Δ*S* or Δ*R*, shaded bars indicate jackknife standard errors of the mean; in experiments with continuous predictors, shaded bars are jackknife standard errors of a running average of the mean (window size = 15°, overlap = 14°). Bars in bar plots are 95% confidence intervals on the parameter estimates. The raw data are available at https://doi.org/10.5281/zenodo.2544946. DOG, derivative of Gaussian function; Δ*R*, delta response; Δ*S*, delta stimulus; fdr, false discovery rate; rmse, root-mean-square error.

Ten participants were presented with peripheral Gabor stimuli (50% Michelson contrast) oriented randomly between 0° and 165° off vertical (in steps of 15°) and were asked to reproduce the perceived orientation by adjusting the tilt of a response bar (Fig 1A). Data were analyzed after residualizing individual adjustment responses from their orientation-dependent shifts in mean [64], a typical confound that falsely mimics response-related serial dependence [65] (see Methods and Fig 2E and 2F). The biased-corrected behavioral data were then used to compare the influence of past stimuli and responses on current errors. To this aim, we built two competing models. In one model, we predicted errors in the adjustment responses with the difference between the previous stimulus orientation and the current stimulus orientation (delta stimulus [Δ*S*]). In the alternative model, we predicted errors with the difference between the previous decision and response and the current stimulus orientation (delta response [Δ*R*]). In both models, errors were fitted to a derivative of Gaussian function (DoG) in a multilevel modeling framework (see Methods), and the amplitude parameter of the DoG (α) was used to quantify the magnitude of serial dependence and to test its significance.

Serial dependence for the stimulus model Δ*S* was significant (α = 1.32°, *p* < 0.05) and had a peak at 9.95° (Fig 2A and 2B), in keeping with previous reports [30]. When errors were conditioned on previous responses (Δ*R*), serial dependence was also significant and qualitatively larger than the one observed for Δ*S* (α = 1.71°, *p* < 0.01, peak at 14.55°; Fig 2B).

To evaluate the best model of serial dependence, we followed two steps: (1) We assessed which model provided a better fit of participants’ errors by computing the difference in the root-mean-square error (*rmse*) between Δ*S* and Δ*R*, and (2) we compared the ability of each model to predict new data with a cross-validation procedure (see Methods). The model comparison in step 1 favored the Δ*R* model over the Δ*S* model (*rmse*[Δ*S* − Δ*R*] = 0.059, *p* < 0.001, Monte Carlo permutation test, *n* = 1,000), and in the cross-validation of step 2, the performance of the Δ*R* model was considerably higher as compared to the Δ*S* model (*rmse*[Δ*R*] < *rmse*[Δ*S*] = 98.6%; Fig 2C). Thus, both procedures favored the Δ*R* model, showing that conditioning on previous responses increased the explanatory and predictive ability of the model.

### Experiment 2: The effect of previous decisions at fovea

The results of Experiment 1 provided strong evidence that previous responses, residualized from orientation-dependent biases, constitute a more efficient predictor of serial dependence than the relative orientation of consecutive stimuli, suggesting that attractive biases may indeed result from the history of perceptual decisions. One possibility is that decisional traces bias perception more than previous stimuli when the sensory input is highly uncertain [53]. Because orientation sensitivity degrades as a function of eccentricity [66,67], the presentation of Gabors in the periphery of the visual field may have favored decisional biases and lowered the contribution of previous uncertain stimuli. Therefore, it could be hypothesized that when stimulus uncertainty decreases, the physical orientation of previous stimuli becomes a more reliable predictor of perceptual errors compared to the previous decision and response. To address this possibility, we performed a second experiment (Experiment 2) in which the same stimuli of Experiment 1 were presented at the fovea. Although there is evidence of stimulus-induced attractive biases at or near the fovea [26], a direct investigation of the impact of previous responses for central stimuli is lacking. In addition, to further reduce stimulus uncertainty (e.g., to increase orientation discriminability), stimuli were presented with higher spatial frequencies (see Methods) [68].

Following the procedure of Experiment 1, in 10 new participants we compared the contribution of Δ*S* and Δ*R* for centrally presented stimuli. In addition, to rule out any confound due to the comparison of one model—the Δ*S*—built with a discrete predictor against a second model—the Δ*R*—built with a continuous predictor [69], we varied the relative orientation of consecutive stimuli in the range of ±90° with steps of 1°, making the Δ*S* predictor fully comparable with the Δ*R*.

Serial dependence at fovea was significant for both Δ*S* (α = 1.40°, *p* < 0.01, peak at 15.6°) and Δ*R* (α = 1.45°, *p* < 0.01, peak at 19°; Fig 2B and 2D). Contrary to the uncertainty hypothesis, however, model comparison again favored the Δ*R* model (*rmse*[Δ*S* − Δ*R*] = 0.003, *p* < 0.05), which also predicted new data with higher accuracy than the Δ*S* model (75.7%; Fig 2C, lower distribution plot), thus fully confirming the results of Experiment 1.

The first two experiments converged to one crucial point: previous perceptual decisions represent a better predictor of serial dependence than previous stimuli.

### Experiment 3: Contrasting forces on serial dependence

Our model comparison revealed that perceptual decisions and behavioral responses are critical, if not fundamental, for serial dependence in orientation discrimination. In their seminal work, Fischer and Whitney [26] reported sequential effects of stimulus presentation in the absence of prior motor responses, ruling out the contribution of postperceptual effects on serial dependence. However, in their experiment and in similar attempts [29,43], participants were not explicitly instructed to withhold their response, and this may have still promoted postperceptual processing of the stimuli (e.g., in the form of implicit decisions and persisting internal representations). As a consequence, even in the absence of an actual overt motor response, perceptual decisions might have occurred, leading to lingering effects during the interstimulus interval, in turn affecting perception of the subsequent Gabor stimulus. Following this reasoning, we predicted that if serial dependence emerges from previous decisions, then it should disappear when participants are explicitly instructed to ignore the most recent stimulus and to withhold their response and decision.

To test this hypothesis, 15 participants performed an experiment (Experiment 3) similar to Experiment 1 but including 40% of “catch” trials in which no response had to be made. Catch trials were explicitly signaled by a black circle appearing at the same location and time as the response bar in the standard trials, indicating that no response—and therefore no perceptual decision—had to be made. Because perceptual decisions were directly manipulated, and to overcome confounds due to orientation biases, we analyzed serial dependence after both catch and response trials only for Δ*S*.

**Fig 3:**
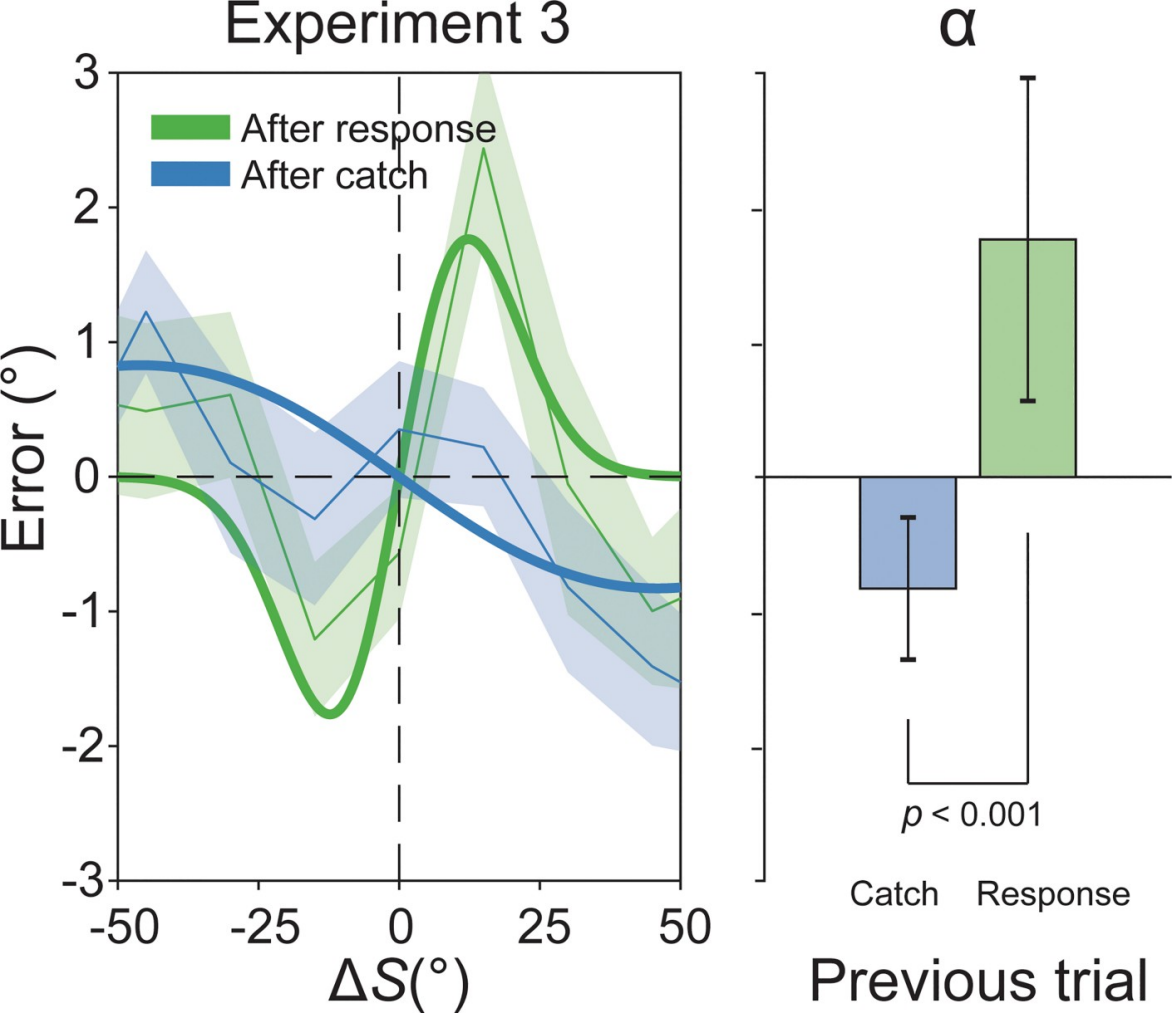
Negative serial dependence after nonresponse trials. Results of Experiment 3 showing that Δ*S* had an opposite effect after catch trials (40%) or response trials (60%). Positive serial dependence was observed after responses (green curve and green shaded bar), whereas negative aftereffects emerged after catch trials (blue curve and blue shaded bar). The magnitude of negative and positive dependencies (α) was significantly different (blue versus green bar, right bar plot). The raw data are available at https://doi.org/10.5281/zenodo.2544946. Δ*S*, delta stimulus.

Although serial dependence for Δ*S* was significant after response trials (α = 1.76°, *p* < 0.01, peak at 12.30°), not only were attractive biases absent after catch trials, but also Δ*S* revealed a repulsive effect on current errors (α = −0.82°, *p* < 0.01, peak at −46°) that was significantly different from the effect after response trials (α[catch]–α[response] = 2.59°, *p* < 0.001; Fig 3), ruling out the possibility that serial dependence simply disappeared because of an intervening stimulus (the black circle).

Confirming our hypothesis, this experiment showed that prior perceptual decisions are fundamental for positive serial dependence, and it revealed that withholding decisions and responses leads to the emergence of repulsive biases, resembling those observed in negative aftereffects [1,4,41].

### Experiment 4: History biases alter perceptual sensitivity

Because the observed positive serial dependence by previous decisions may originate at a later stage than early perceptual processing, one may argue that the reported effect is due to postperceptual biases [30] emerging during adjustment responses and having no direct impact on the quality of a perceptual experience [48]. In order to better characterize the nature of these opposite biases, and before drawing any firm conclusion, in the present experiment (Experiment 4) we investigated whether past decisions and stimuli act directly on perception and on the sensitivity of the perceptual system. To this aim, we abandoned the adjustment paradigm and employed a combination of forced-choice tasks, which have been proposed as a more direct measure of perception [29,30].

**Fig 4:**
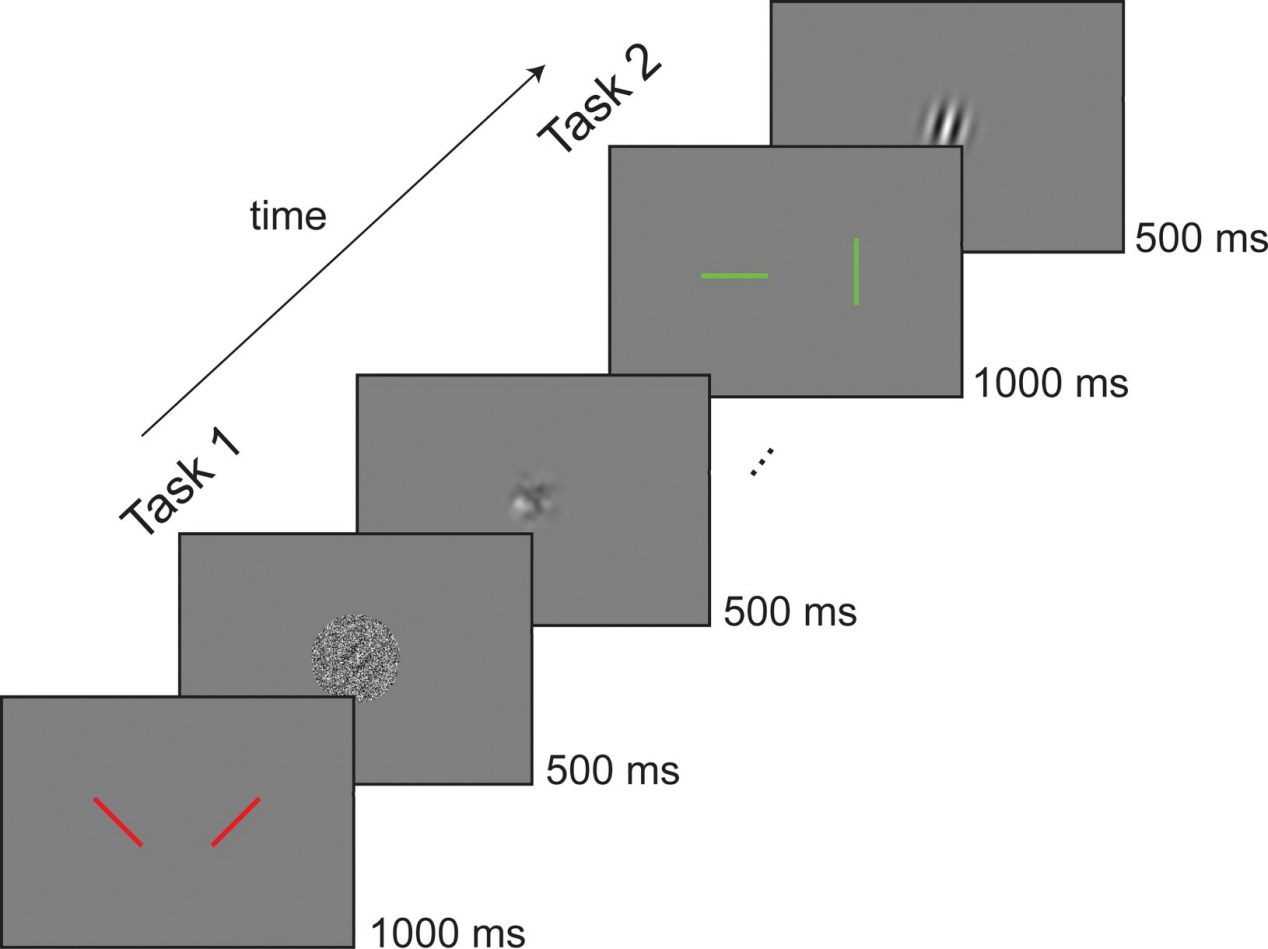
Example of one trial in the dual task of Experiment 4. In the first task of each trial, participants were cued by two oblique red bars to report the left versus right orientation of an upcoming Gabor presented with different SNRs (0%, 25%, 75%, 25% is shown as a representative condition) and followed by a noise mask. In the second part, green cardinal lines cued participants to report the vertical/horizontal deviation of the orientation of a second Gabor. SNR, signal-to-noise ratio.

Fourteen new participants were asked to perform a dual task with sequences of Gabor stimuli presented at fovea (see Methods and Fig 4). In the first task, they had to discriminate the left or right oblique orientation of a Gabor followed by a noisy mask (45° versus 135°, one-alternative forced-choice [1AFC] symmetric task [70]). In the second task, they had to report whether an ensuing Gabor of variable orientation (±2, 5, 15, or 40° off the left or right oblique, positive toward vertical) was more tilted toward the vertical or the horizontal (recoded as a “Yes/No” vertical task, see Methods). In half of the Yes/No trials, the reference oblique (i.e., the oblique bisecting vertical/horizontal) was the orientation presented or reported in the 1AFC task (Congruent condition). In the other half, the reference oblique was the opposite (Incongruent condition). Gabors in the first 1AFC task were presented with different signal-to-noise ratios (SNRs = 0%, 25%, and 75%), with one condition containing only noise (i.e., SNR = 0%).

The combination of these forced-choice tasks allowed for a direct test of the effect of previous stimuli and decisions on the discriminability and appearance of present sensory events. In adherence with our previous results, we formulated two testable hypotheses: (1) If past stimuli repel perception, then the sensitivity to detect deviations from an oblique orientation should increase if the oblique is presented in a preceding task (e.g., in Congruent trials). (2) If previous decisions attract current perception, then the net effect of a decision (i.e., in the absence of sensory signal, SNR = 0%) would be to decrease the sensitivity to small orientation changes by integrating present stimuli close to the previous decisional outcome (e.g., congruent to the reported orientation).

**Fig 5:**
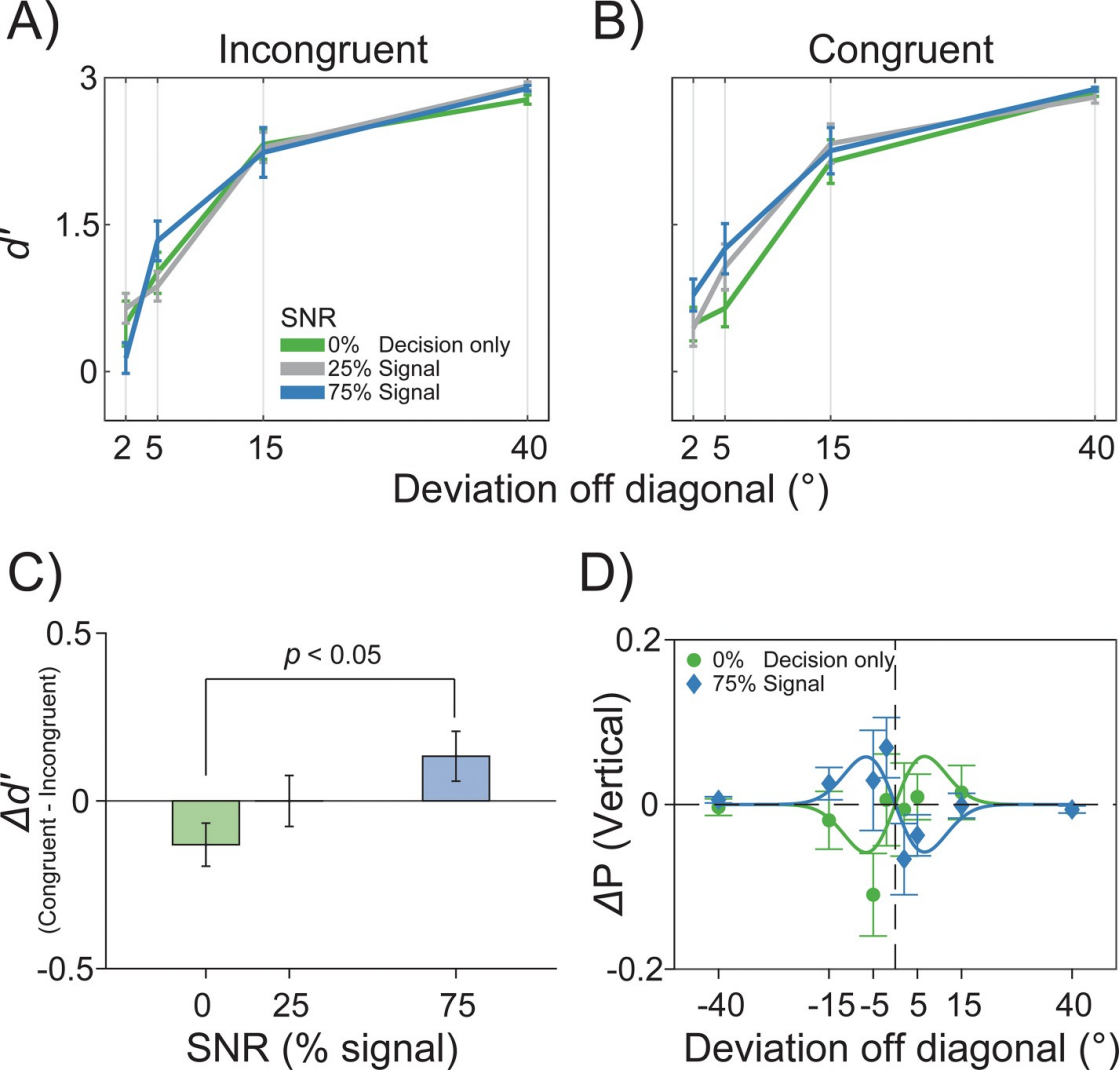
Results of the Vertical/Horizontal task in Experiment 4. (A–B) Perceptual sensitivity (*d*’) as a function of the increasing deviation of the Gabor from oblique and of the SNR in the preceding Left/Right task, for both the Incongruent (A) and Congruent (B) conditions (see Methods for a detailed description). (C) The difference in the overall *d*’ between Incongruent and Congruent trials (collapsed over deviations) revealed the effect of previous stimuli (75% SNR) and past decisions in the absence of stimuli (0% SNR): Previous stimuli increased the sensitivity to changes around their orientation; previous decisions had the opposite effect. (D) The raw average proportions of “vertical” responses for SNR 0 and 75% (Incongruent minus Congruent) showed a DoG-like pattern of opposite sensitivity changes. The raw data are available at https://doi.org/10.5281/zenodo.2544946. DoG, derivative of Gaussian function; SNR, signal-to-noise ratio.

To address these hypotheses, we modeled participants’ sensitivity (*d*’) in the Yes/No task as a function of the stimulus deviation toward cardinal axes and of the SNR in the preceding 1AFC task. Incongruent trials were treated as a control condition, since stimuli and decisions 90° away from the present orientation should have no effect [29]. As expected, in the Incongruent condition (Fig 5A) a multilevel linear model revealed a significant intercept (*β*_0_ = 0.78, *p* < 0.001) and a significant slope for Deviation (the increase of *d*’ with the increasing deviation from oblique, *β*_1_ = 0.05, *p* < 0.001) but no effect of the SNR and no interaction Deviation × SNR (all *p* > 0.05). In the Congruent condition (Fig 5B), however, in addition to the intercept (*β*_0_ = 0.60, *p* < 0.01) and slope for Deviation (*β*_1_ = 0.06, *p* < 0.001), the model revealed also a significant main effect of SNR (*β*_2_ = 0.005, *p* < 0.05) with no interaction Deviation × SNR (*β*_3_, *p* = 0.23). The overall modulation of *d*’ by the SNR was then compared to a baseline *d*’ in the Incongruent control condition (see Methods). This baseline-corrected sensitivity showed a decreased *d*’ for decision-only trials (SNR = 0%, Δ*d*’ = −0.13) and an increased *d*’ after high-SNR stimuli (SNR = 75%, Δ*d*’ = 0.13), with a significant difference between the two (paired *t* test, *p* < 0.05; Fig 5C).

To further characterize this difference, we performed a final analysis on the raw proportions of vertical responses over the whole range of orientations tested in the Yes/No task. By subtracting the two Congruent conditions of interest (SNR = 0% and 75%) from the baseline (Incongruent condition), the resulting difference in proportions of vertical responses Δ*P*(Vertical) showed an evident DoG-like pattern: after decision-only trials (SNR = 0%), participants responded more often “vertical” when the tilt was slightly horizontal and less often “vertical” when the tilt was slightly vertical; conversely, after high-SNR stimuli this pattern was reversed, suggesting a more sensitive discrimination of the vertical/horizontal direction than in the baseline (Fig 5D). The two conditions were well fitted by the multilevel conditional DoG function used in Experiment 3 (Eqs 3 and 4), with a significant positive amplitude for decisions (α = 0.058, *p* < 0.05), a trend for a negative amplitude for stimuli (α = −0.057, *p* = 0.06), and a significant difference between the two (0% SNR − 75% SNR = 0.11, *p* < 0.01).

This intriguing pattern confirmed the opposite effects of previous decisions and stimuli in the absence of any potential confound due to postperceptual processes and adjustment procedures. Furthermore, it demonstrated that history biases act directly on perceptual sensitivity: previous decisions (in the absence of sensory signals) attract future perceptual representations, decreasing the discriminability of consecutive stimuli; strong sensory signals repel future stimuli, aiding their differentiation.

### Experiment 5: Testing a Two-process model of serial dependence

The opposite effect of past stimuli and perceptual decisions is in sharp contrast with the idea of serial dependence as a low-level visual mechanism [26]. In line with the assumption of low-level continuity fields, indeed, serial dependence would originate from changes in the response properties of early visual neurons and should be immune to neuronal computations at later, decisional stages. However, our results revealed two mechanisms regulating serial dependence: one repulsive, after the mere exposure to sensory stimuli, and the other attractive, observed after perceptual reports. These contrasting forces on visual perception suggest that positive and negative carryover effects may originate at different stages of the processing stream [30,56].

**Fig 6:**
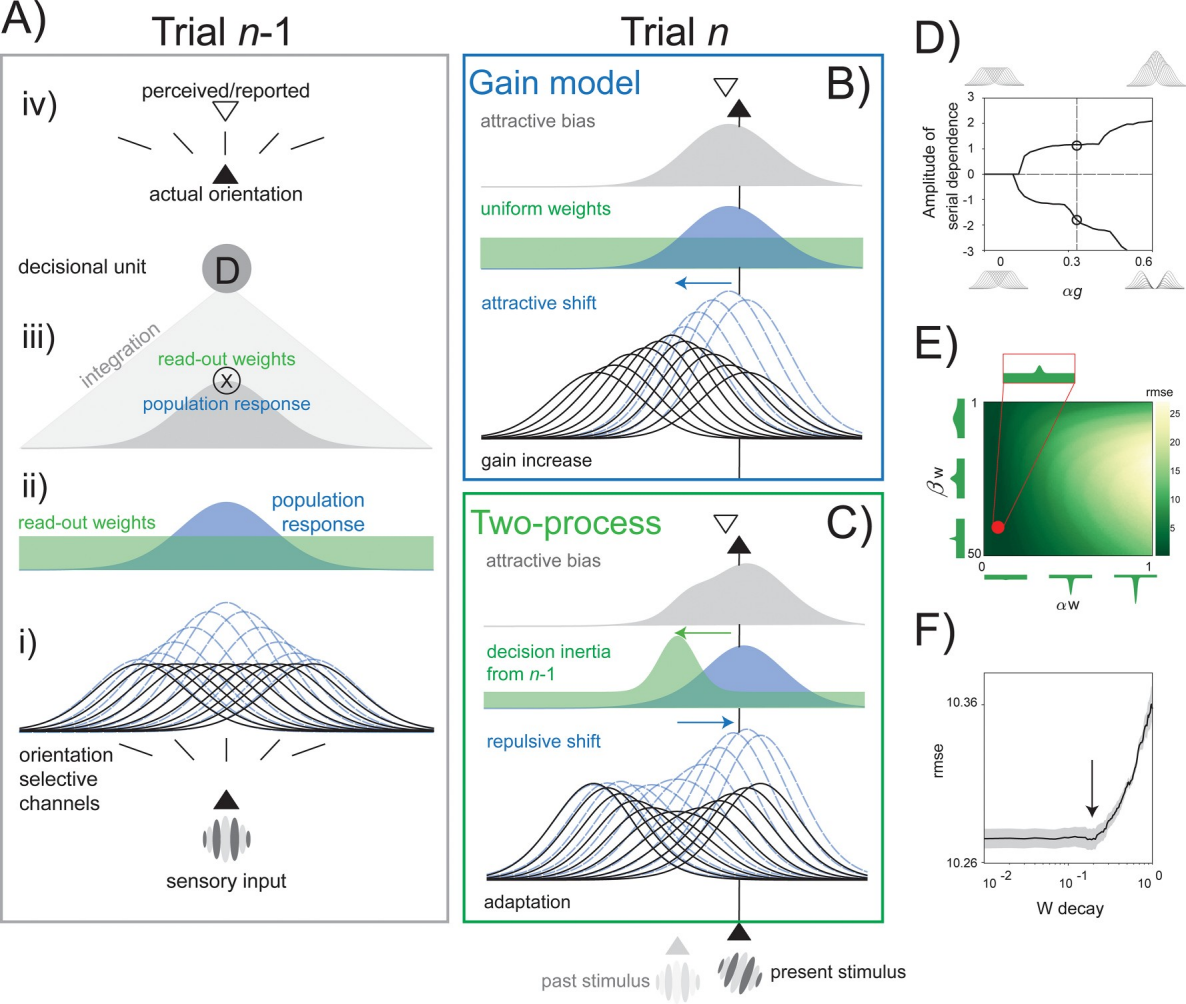
Two data-generating models of serial dependence. (A) The general model architecture and the response to a first vertical orientation (trial *n*-1). From bottom to top: (i) a subset of low-level orientation-selective units with individual tuning curves around their preferred orientations (black curves) and their individual response to the input stimulus (blue dashed curves); (ii) population response profile (blue distribution) characterized by individual responses of low-level units and a uniform distribution of decisional readout weights (green distribution) in the absence of stimulus and decision history; (iii) the decoding of sensory evidence from a decisional unit computed through a weighted average of the population response multiplied by decisional weights (gray distribution); (iv) in a noise-free example and in the absence of stimulus and decision history, the decoded orientation relayed to the final response stage (white triangle) corresponds to the stimulus in input (black triangle). (B) The Gain model tested on trial *n* with an input orientation of 20°. A gain change (black curves) attracts the new stimulus toward the orientation presented before, altering the population response profile (blue dashed curves). Decisional weights are uniform and constant in this model (green distribution). (C) The behavior of the Two-process model in trial *n*. A decisional template formed in the previous trial infiltrates the processing of new stimuli by altering the readout weights of low-level activity (nonuniform green distribution). This way, decisional traces contrast the repulsive effect of low-level adaptation (blue curves and distribution), leading to positive serial dependence. (D–F) Optimization of the critical parameters in the Gain and Two-process models: The evaluation of the impact of αg (the gain factor) in modulating the amplitude of positive serial dependence (positive values on the *y* axis, obtained with Eq 9) or adaptation (negative values in the *y* axis, obtained with Eq 10, see Methods) (D); the optimization of the decisional template by independently varying its amplitude αw and width βw (βw is varied in logarithmic steps), minimizing the rmse with the observed shape of serial dependence in our Experiment 1, 2, and 3 (E); the optimal W decay (forgetting factor) was estimated by minimizing the model error with respect to the single-trial performance of our participants in Experiments 1 and 2 (F). rmse, root-mean-square error.

To explore the plausibility of multiple stages of serial dependence, we defined two data-generating models (Fig 6) that implemented serial dependence either as a consequence of low-level processes (Gain model) or as a combination of opposite forces exerted by mechanisms at different stages (Two-process model). In both cases, perception was modeled as an encoding–decoding process performed by a hierarchy of units based on recent models of visual processing [54,71–73].

The model’s architecture (Fig 6A) included a layer of low-level units resembling a population of orientation-selective neurons, a higher-level decisional unit, and a final response stage. The presentation of an oriented stimulus elicited a population response in which the contribution of each low-level unit depended on the distance between the stimulus and the unit’s preferred orientation. The decision unit then decoded and integrated the population profile into a final response (the reported orientation). The decoding stage occurred through a set of weights, mediating the readout of the decision unit from each orientation-selective cell [54]. Thus, orientation discrimination was modeled as a two-stage process: an initial response by a population of perceptual units and a later weighted decoding by a higher-level decisional unit.

In the Gain model (Fig 6B), serial dependence was produced by a gain change in the response properties of orientation-selective units [26]. Exposure to a tilted stimulus increased the sensitivity of low-level units coding the same and nearby orientations, and this in turn biased the population response to an incoming stimulus toward the orientation coded in the past. In this model, the decision unit decoded the population profile with uniform and constant weights; thus, serial dependence only emerged from trial-by-trial gain modulations at the level of orientation-selective neurons.

In the Two-process model (Fig 6C), serial dependence resulted from the interaction between low-level adaptation mechanisms and changes in decisional weights. Contrarily to the Gain model, exposure to oriented stimuli inhibited the response of low-level units centered onto the same and nearby orientations. This, in turn, biased the population response to an incoming stimulus away from the present orientation [74,75], resembling negative aftereffects observed after both brief and prolonged adaptation periods [76–78]. The critical aspect of the Two-process model was the plasticity at the level of decisional weights. In this model, previous decoding templates persisted, generating a nonuniform distribution of weights with maxima at/near the orientation decoded and reported in the recent past. This way, opposite carryover effects were modeled at different stages of the processing stream, with negative aftereffects arising from low-level adaptation and attractive biases emerging from decisional traces. Because the reweighting process favored the readout from orientation channels that were more informative in the recent past, this effect compensated for trial-by-trial adaptation at the lower level, leading to the positive serial dependence observed after behavioral responses. The reweighting process was triggered by the requirement—and execution—of a behavioral response, and therefore, no weight update followed the mere exposure to irrelevant stimuli.

**Fig 7:**
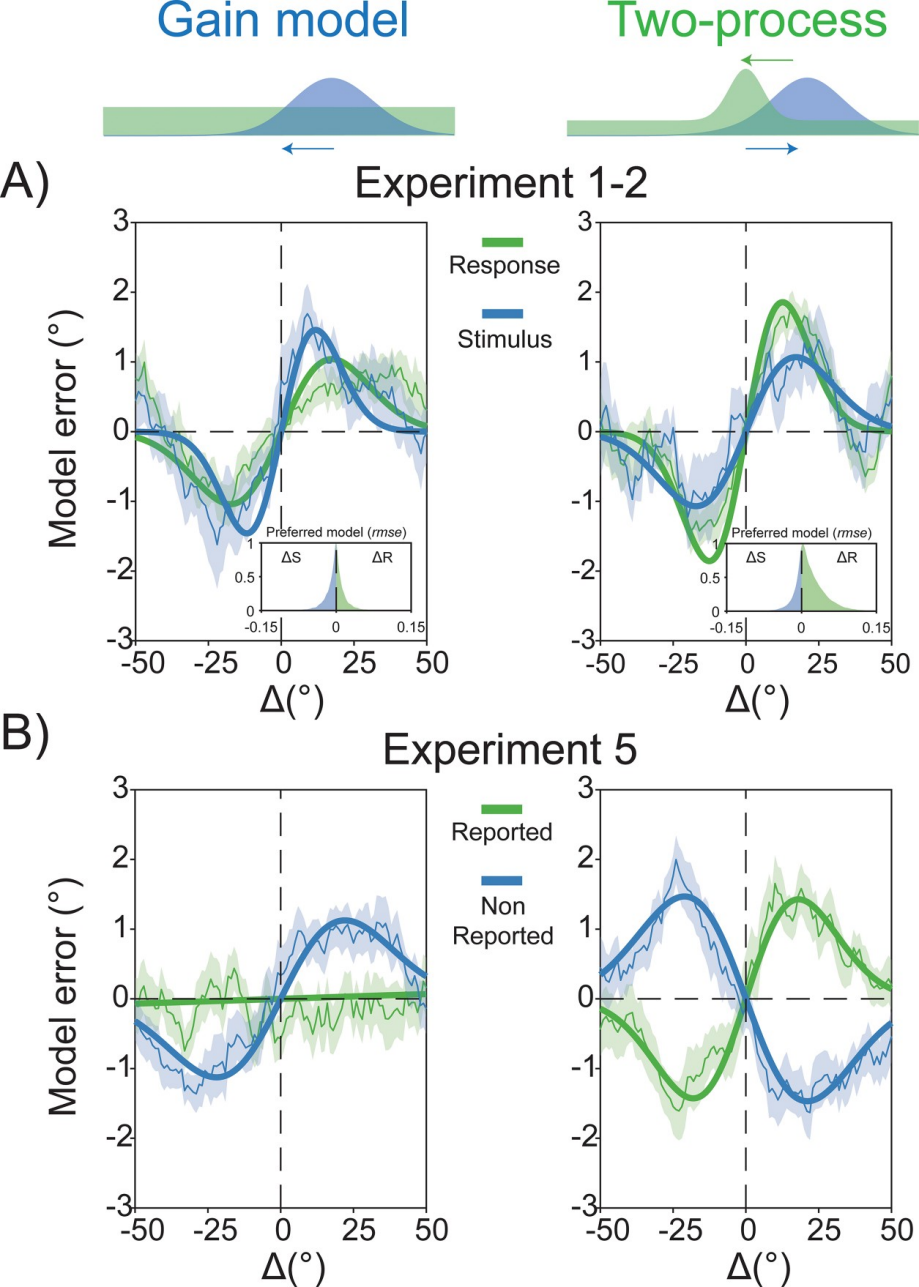
Predictions of the two data-generating models. (A) Compared to the Gain model (left plot), the Two-process model (right plot) better approximated the general pattern of results observed in Experiments 1 and 2, reproducing the observed improvement in model fit when considering Δ*R* instead of Δ*S* (inset plots of rmse). (B) Predictions of the two models in the context of Experiment 5. The Gain model (left plot) showed consistent dependencies on the last nonreported stimulus in the sequence, with no persisting effect of the orientation reported in the past. The Two-process model (right plot) revealed the opposite effect of repulsive adaptation for nonreported stimuli and attractive serial dependence for the perceptual decision and related stimulus one trial in the past. Δ*R*, delta response; Δ*S*, delta stimulus; rmse, root-mean-square error.

The Two-process model provides a compelling account for the coexistence of both positive and negative dependencies in visual perception [30,56]; however, its plausibility and performance had to be evaluated and tested against the Gain model. To this aim, we first compared the two models in a simulation of behavioral errors for the trial sequences used in Experiments 1 and 2. In this context, the Two-process model closely resembled the observed patterns of serial dependence, and it reproduced the superior predictive ability of Δ*R* over Δ*S* that we characterized in our first two experiments (Fig 7A). As a more stringent test, we then compared the predictions of the two models with the true pattern of errors made by participants in a new experiment. In this experiment (Experiment 5; Fig 1B), 15 participants were presented with a sequence of Gabors in each single trial and were asked to report the orientation of the last stimulus after the sequence ended (see Methods). Because previous decisions were always interleaved with a sequence of new stimuli before the next to-be-reported orientation, this paradigm offered an ideal situation in which previous stimuli and decisions were dissociated and both positive and negative serial dependence could emerge. To evaluate the predictions made by the two models in this experimental context, we ran a simulation of Experiment 5 (*n* = 10,000), and we conditioned the error generated by both models on previous stimuli after perceptual decisions (the reported orientation in the previous trial) or in the absence of decisions and responses (the last nonreported stimulus in the sequence).

**Fig 8:**
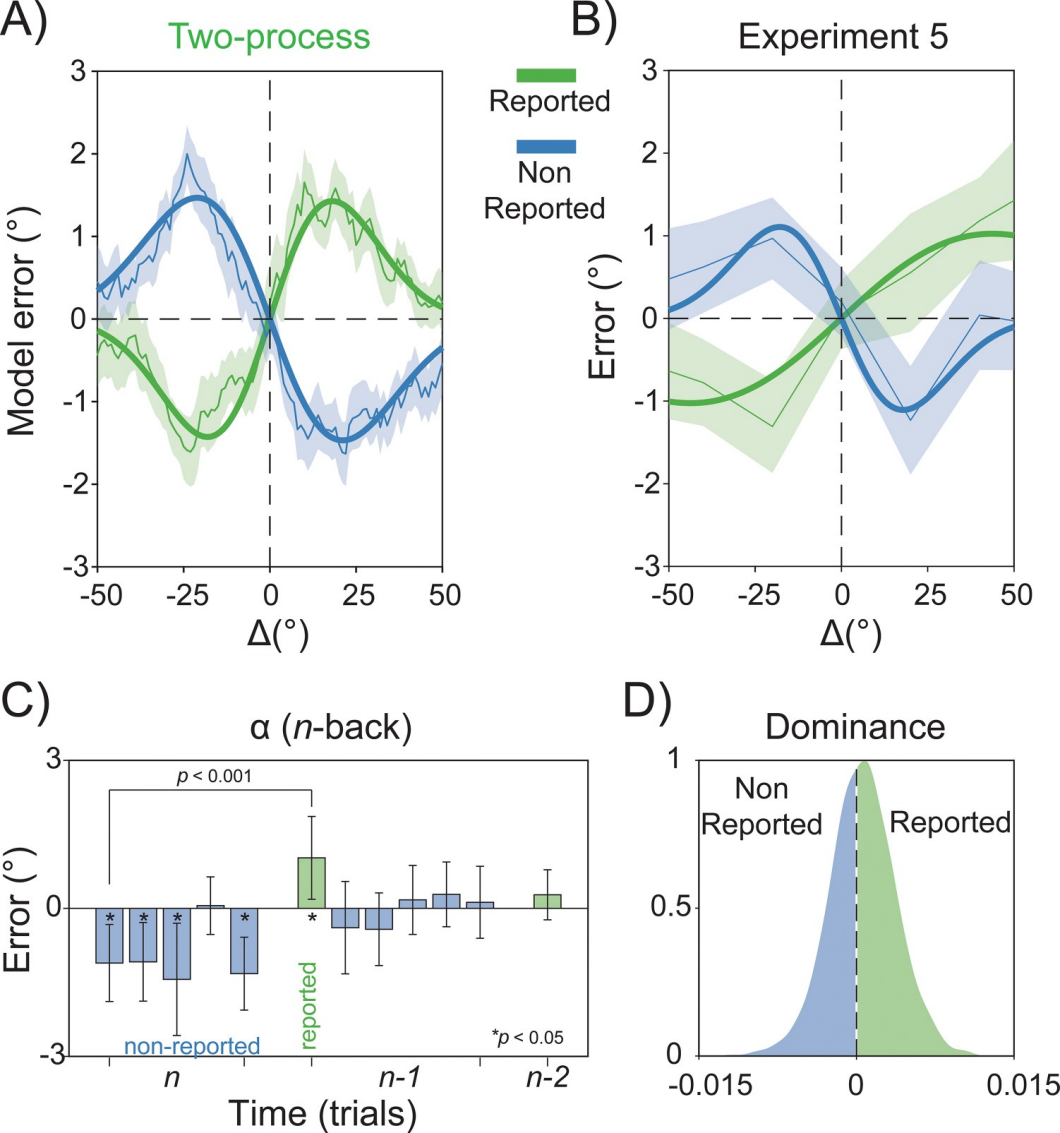
Opposite effects of previous stimuli and decisions on perception. (A–B) In excellent agreement with the predictions of the Two-process model (A), the observed results of Experiment 5 (B) revealed the opposite effects of previous stimuli (nonreported) and perceptual decisions (reported) on current perception. (C) Almost all stimuli in the last nonreported sequence exerted a repulsive effect. (D) In the context of this experiment, where past decisions and stimuli were orthogonal, opposite biases from previous decisions and sensory signals dominated single perceptual reports with almost equal contribution. The raw data are available at https://doi.org/10.5281/zenodo.2544946.

As expected, the Gain model predicted positive serial dependence for the preceding stimulus in the sequence but no persisting influence of the orientation reported in the previous trial, i.e., of the previous perceptual decision (Fig 7B). On the contrary, previous stimuli and perceptual decisions had opposite effects on the errors generated by the Two-process model: although adaptation at the low level caused repulsive biases, prior decisional weights attracted the representation of incoming stimuli (Fig 7B). In order to select which data-generating process provided a better approximation of the mechanisms underlying serial dependence, we compared the outcomes of both models with the actual data from Experiment 5. The behavioral results resembled almost exactly the predictions made by the Two-process model (Fig 8A and 8B). At odds with the Gain model, a significant negative serial dependence was observed in response to the previous—nonreported—stimulus in the sequence (α = −1.10°, *p* < 0.01, peak at −17.9°), whereas positive carryover effects emerged for stimuli reported in the preceding trial (α = 1.02°, *p* < 0.05, peak at 43.75°), and the magnitude of the two dependency effects was significantly different (α[Reported] − α[Nonreported] = 2.13°, *p* < 0.001; Fig 8B and 8C). Furthermore, the effect of nonreported stimuli was generally repulsive (Fig 8C), whereas opposite biases from previous decisions and past stimuli dominated single perceptual reports with almost equal contribution (dominance for reported stimuli = about 57%; see Methods and Fig 8D).

This clear-cut result provided strong evidence in favor of a hierarchical view of serial dependence, and in addition, it showed that decisional traces affect current perceptual decisions even when interleaved with new stimuli.

### Experiment 6: Stimulus strength and decisional traces

The fact that the lingering effect of perceptual decisions persisted across time, even after streaks of intervening stimuli, is in line with the idea that positive serial dependence emerges from the inertia of a high-level decision unit, which operates in a stimulus-independent manner. An interesting question is therefore whether decisional traces, once formed, become mere abstractions of a (biased) sensory experience or still preserve some low-level properties of the sensory evidence upon which they are based.

In the present experiment (Experiment 6), we focused on the role of stimulus contrast on serial dependence by perceptual decisions. The aim was 2-fold: (1) to evaluate the contribution of the actual and previous sensory evidence, as determined by the stimulus contrast, and (2) to further address whether decisional traces only rely on perceptual reports (the orientations confirmed with the response bar) or their impact varies with the strength of the previously decoded stimulus. More specifically, if positive serial dependence requires weak sensory signals and the mere absence of adaptation [29,43], then it should increase for streaks of low-contrast stimuli and should decrease (or even turn negative) for streaks of high-contrast stimuli; contrarily, if attractive biases are due to persistent traces in decisional circuits that counteract adaptation, then the stronger the perturbation of the circuit was (e.g., when decisions were made on high-contrast stimuli), the larger the actual attractive bias will be.

**Fig 9:**
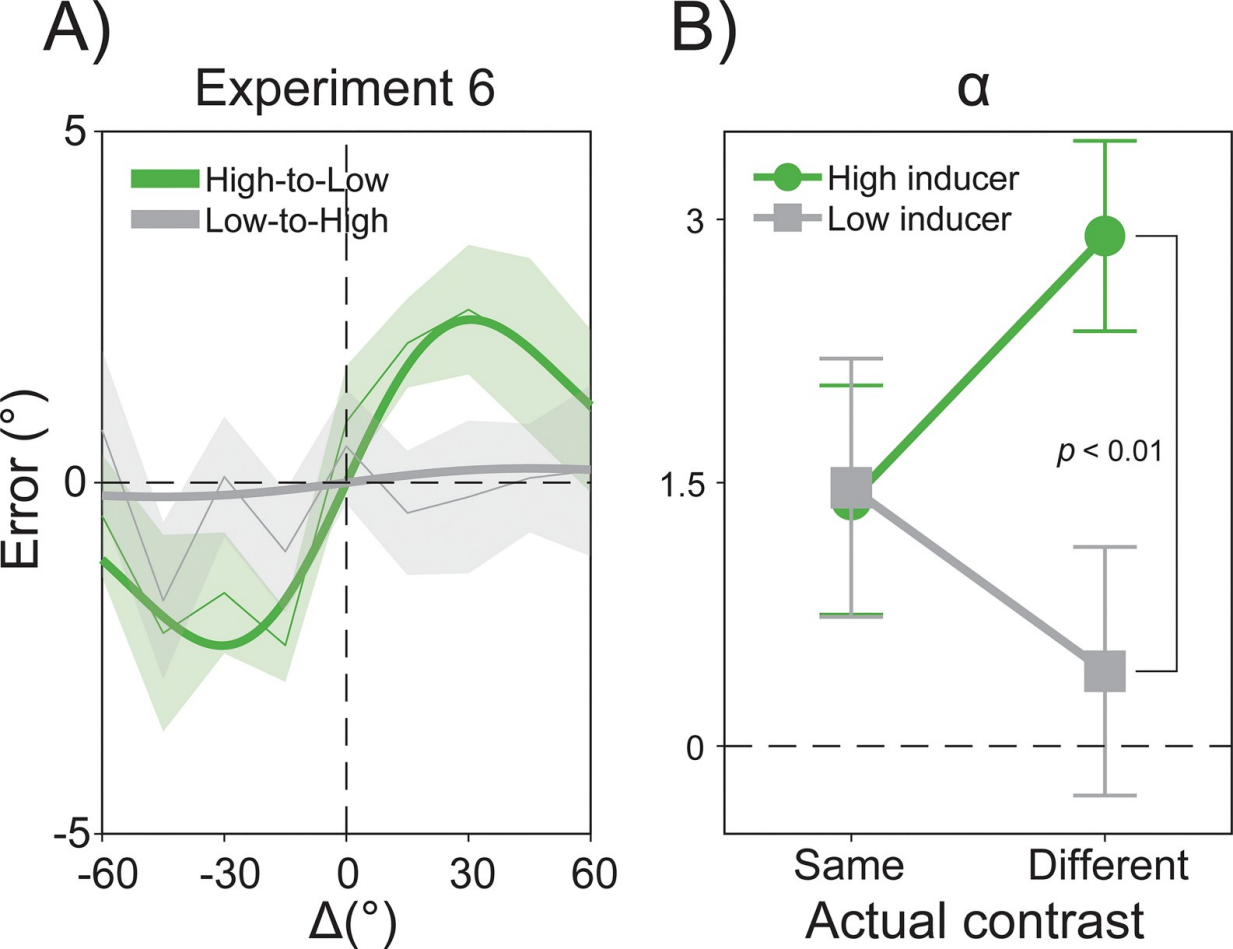
Results of Experiment 6. (A) High-contrast inducer stimuli increased the impact of previous decisions on the processing of low-contrast stimuli. (B) The interaction Inducer Contrast × Actual Contrast. The raw data are available at https://doi.org/10.5281/zenodo.2544946.

Fifteen participants performed the same orientation adjustment task as in Experiment 1, with stimuli of high (75%) and low (25%) contrast randomly intermixed across trials. In a first multilevel model, we compared the effect of switching between low and high stimulus contrast by extracting the α parameter when a low-contrast inducer was followed by high-contrast stimuli and vice versa when a high-contrast inducer was followed by low-contrast stimuli (Fig 9A). This analysis revealed a strong and significant serial dependence for Δ*S* only in the high-to-low condition (α = 2.32°, *p* < 0.001, peak at 30.6°) but no attractive biases in the low-to-high condition (α = 0.20°, *p* > 0.05), with a significant difference between the two (high-to-low minus low-to-high = 2.11°, *p* < 0.01).

To further characterize this interaction between the contrast of previous and present stimuli, we then estimated the magnitude of serial dependence at the single-subject level in order to compare four conditions of interest in a full factorial design. Individual α values were submitted to two-way repeated-measures ANOVA with factors Inducer Contrast (Low versus High), Actual Contrast (Same [as the inducer] versus Different), and their interaction. The ANOVA yielded a significant interaction Inducer Contrast × Actual Contrast (F_(1,14)_ = 6.03, *p* < 0.05, *η*^2^ = 0.3) driven by the significant difference between high-to-low- and low-to-high-contrast serial dependence (*p* < 0.01, Bonferroni correction; Fig 9B). Thus, decisional traces are stronger after high-contrast stimuli, and their attractive influence increases in relation to weak stimuli.

### Experiment 7: Generalization of serial dependence to ensemble coding

**Fig 10:**
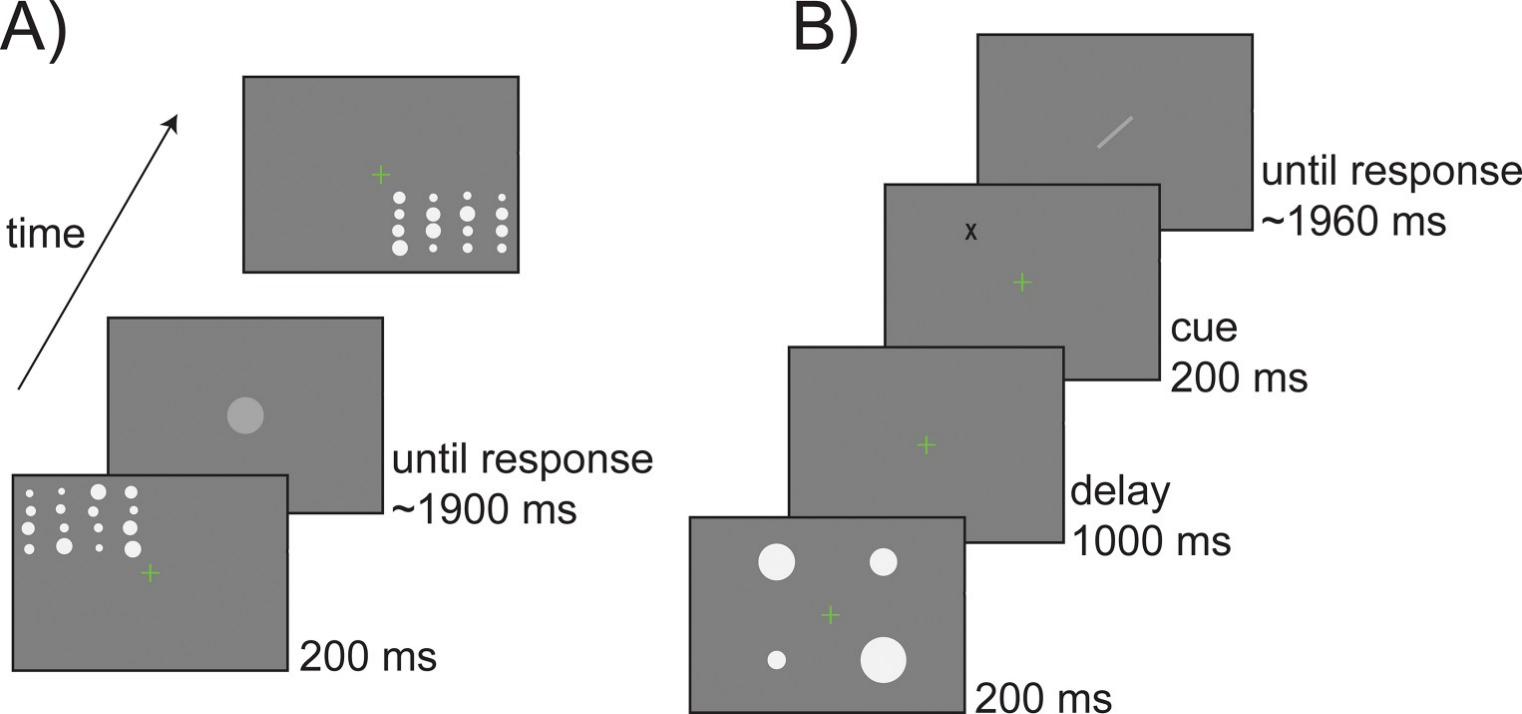
(A) Example of one trial in the ensemble coding task of Experiment 7. Participants viewed an ensemble of 16 dots in one of the four quadrants and had to report the average size by adjusting the size of a response dot. (B) Example of one trial with four items in the working memory task of Experiment 8. Participants had to memorize the size of each dot and reproduce the diameter of the dot indicated by a retro-cue (black X). The diameter was reported by adjusting the length of a central bar, which was randomly oriented on each trial.

To generalize our findings to domains other than orientation perception, we performed a further experiment (Experiment 7) in which the task required participants to reproduce the average size of an ensemble of circles (see Methods and Fig 10A). Perceptual averaging, also defined as statistical representation or ensemble coding [79,80], refers to the ability of our perceptual system to extract global statistics, such as the mean or variability, from sets of similar stimuli, thus providing a coarse gist of complex visual scenes. It has been recently shown that ensemble coding occurs in space as well as in time and, consequently, the perceived statistics of an ensemble depends on its global properties in the immediate past [31]. These results were discussed as evidence of a spatiotemporal continuity field in visual perception that promotes objects stability and coherence over time. However, based on the evidence reported thus far, it is plausible that serial dependence in summary statistic is due to the same persistence of decisional templates that we have characterized for orientation processing.

**Fig 11:**
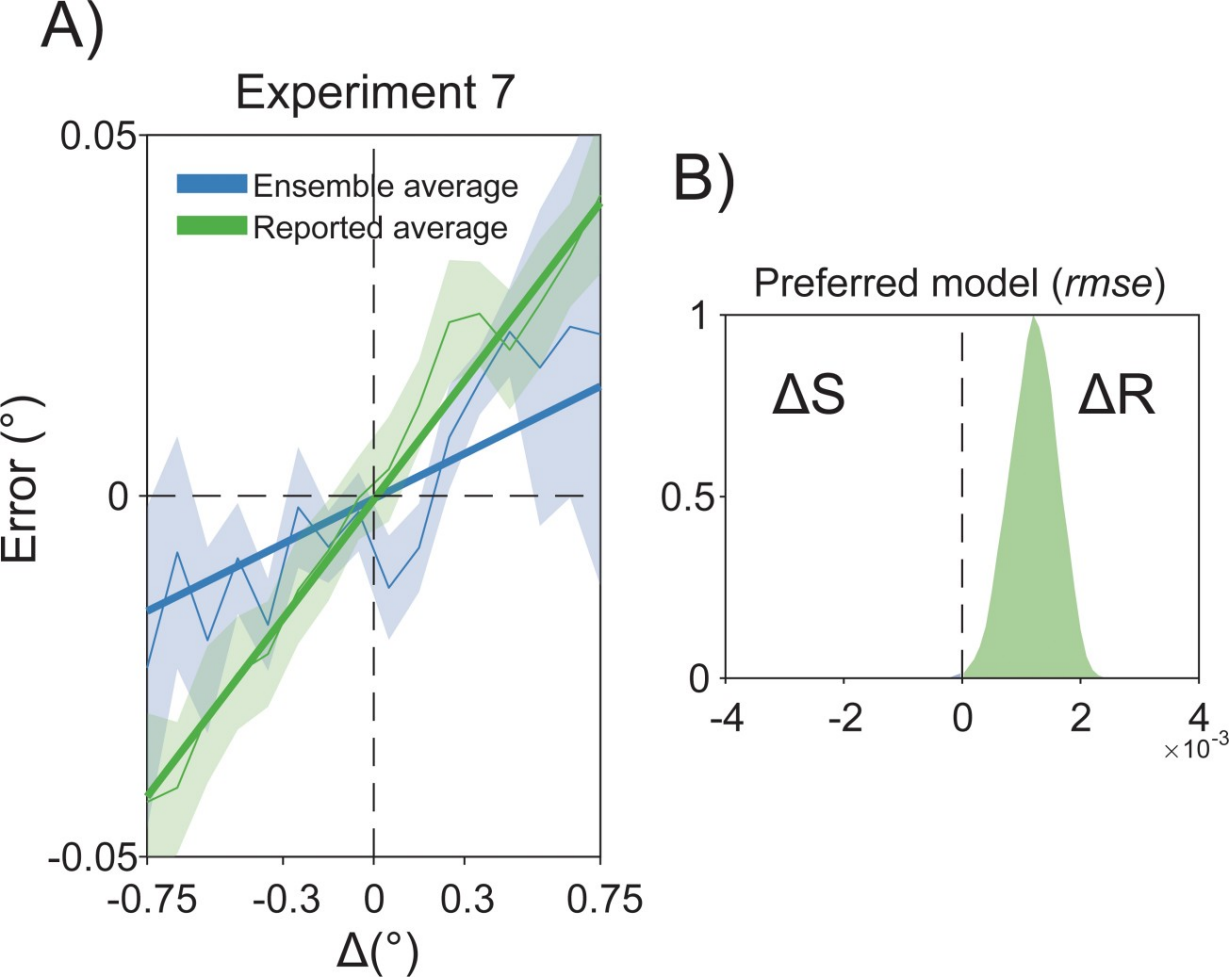
The effect of Δ*S* and Δ*R* in the ensemble coding task of Experiment 7. (A) Judgments of the average size were consistently biased by previous responses (Δ*R*, green lines and green shaded bars) more than the past ensemble average size (Δ*S*, blue lines and blue shaded bars). (B) Consistently with all of our experiments, the Δ*R* model had higher predictive ability than the Δ*S* model. The raw data are available at https://doi.org/10.5281/zenodo.2544946. Δ*R*, delta response; Δ*S*, delta stimulus; *rmse*, root-mean-square error.

To test this hypothesis, we predicted errors in the reported average size of 15 new participants with two different multilevel linear models, following the approach of our Experiments 1 and 2. In one model (Δ*S*), we used the difference between the average size of the previous and present ensemble as the dependent variable. In the other model (Δ*R*), we used the difference between the reported average size in the last trial and the current average size of the ensemble. Before model comparison, errors were corrected for several confounds that typically affect size adjustment responses (see Methods). The Δ*R* model was superior to the Δ*S* model in the overall fit (Akaike information criterion [AIC] for Δ*S* = 1,484; AIC for Δ*R* = 1,399; *p* < 0.001, theoretical likelihood ratio test [TLRT]) and predicted new data with higher accuracy (99.9%; see Fig 11B). Moreover, previous responses were significantly associated with errors of reported size (*β*_1_ = 0.054, *p* < 0.001), whereas in the Δ*S* model the effect of previous stimuli was quantitatively smaller (*β*_1_ = 0.02, *p* < 0.01) and significantly different from the one of previous responses (*β*_1_[Δ*R*] − *β*_1_[Δ*S*] = 0.034, *p* < 0.001). This result showed that the influence of previous responses/decisions has a stronger impact on perceptual averaging than the average size of previous ensembles (Fig 11A).

### Experiment 8: Previous decisions versus memory traces

As an alternative to the Two-process account, one can argue that positive history biases arise during the retention of perceptual or decisional information in a temporary memory storage where persistent traces may interfere (i.e., the working memory account [30,48]). Although our results appear consistent with decisional inertia in reading-out sensory information, as a final step we found it worthwhile to rule out this alternative interpretation. To this aim, in two versions of this final experiment (Experiment 8a and 8b), we evaluated the persistence of previous decisions when visual working memory was filled with new items (see Methods).

Participants were presented with two (*n* = 6) or four (*n =* 10) circles at the beginning of each trial and were asked to memorize the diameter of each single circle. A spatial retroactive cue during the retention interval indicated which circle’s diameter had to be reproduced with an adjustment line (Fig 10B). With this method, visual working memory was constantly filled with new and more recent representations (e.g., the competing stimuli on each display), thus potentially wiping out or at least partly overwriting representations in working memory that might be responsible for positive biases in perception due to recent history. If serial dependence is due to representations in working memory, then more recent traces should have stronger impact on perceptual errors than previous decisions.

**Fig 12:**
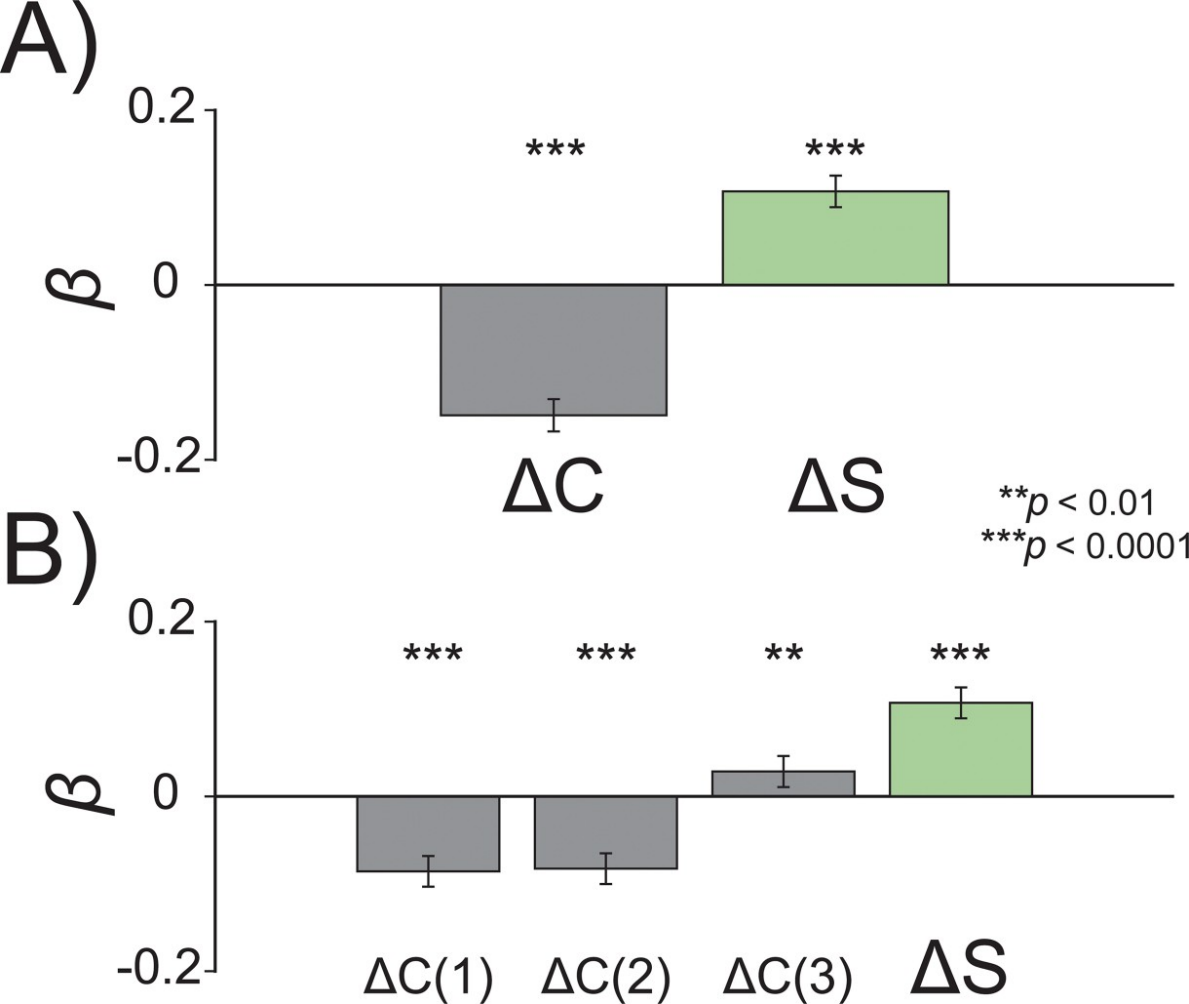
Results of the working memory task with two (A) and four (B) items in Experiment 8: whereas competitive items in memory (ΔC) have a repulsive effect on perceptual reports, the attractive impact of previous stimuli (ΔS) and of the related perceptual decisions was unaffected by the memory manipulation. The raw data are available at https://doi.org/10.5281/zenodo.2544946. ΔC, competitor(s) relative size; ΔS, delta stimulus.

To test this hypothesis, we predicted errors in the reported diameter size of the retro-cued circle as a function of the competitor(s) relative size (Δ*C*) and of the Δ*S*—i.e., the difference between the previous and the current target circle’s size. In the experiment with two circles (Fig 12A), a multilevel linear model revealed a significant repulsive effect of Δ*C* (*β*_1_ = −0.14, *p* < 0.0001) and an opposite, attractive effect of Δ*S* (*β*_1_ = 0.10, *p* < 0.0001). Similarly, in the experiment with four circles (Fig 12B), the effect of Δ*C* was repulsive for the two competitors that lay closest to the retro-cued circle (*β*_1_ = −0.085, −0.082, both *p* < 0.0001) but attractive for the distant competitor (*β*_1_ = 0.028, *p* < 0.01), while a strong effect of previous target was still present (*β*_1_ = 0.10, *p* < 0.0001). Finally, we also performed a model comparison similar to that of Experiments 1, 2, and 7, using Δ*R* instead of Δ*S*. In both experiments, the model with Δ*R* explained the data better than the model with Δ*S* (experiment with two circles: AIC for Δ*S* = 4,525; AIC for Δ*R* = 4,477; *p* < 0.001; experiment with four circles: AIC for Δ*S* = 9,481; AIC for Δ*R* = 9,448; *p* < 0.001, TLRT), thus further confirming one of the main findings of this work.

These results are in line with previous studies showing strategic and repulsive interactions between items in working memory [81], and they reveal a persistent effect of previous decisions/responses that is independent of the recent content and relational representations in a visual working memory storage.

## Discussion

The content of visual perception is strongly permeated by information lingering from the past. In the present study, we demonstrated that vision is shaped by two contrasting forces arising from the history of physical stimuli and perceptual decisions. Our work, based on multilevel modeling and cross-validation approaches, provides a thorough and comprehensive characterization of visual serial dependence from which we derived the following conclusions: (1) There are two contrasting forces that, at any given moment, interact in shaping our perception, biasing the appearance of visual stimuli toward or away from the past; (2) repulsive forces, resembling low-level adaptation, dominate after stimuli that require no perceptual decision; and (3) attractive forces on perception emerge during serially dependent perceptual decisions.

Our primary aim was to compare the ability of previous stimuli and responses to predict participants’ errors in orientation adjustment tasks. This comparison was performed in the first two experiments (and supported by additional analysis in Experiments 7–8) and showed, without ambiguity, that serial dependence in visual perception is explained by the history of perceptual decisions that are required for behavioral responses. Previous responses outperformed past stimuli in predicting participants’ errors for both peripheral and foveal stimuli. This indicated that response-induced history biases are independent of stimulus uncertainty and persist even when the perceptual resolution of stimuli increases.

In a following step, we hypothesized that serial dependence occurs exclusively after stimuli passed through a complete perceptual and decisional processing. The results of Experiment 3 revealed the opposite effects of stimulus and response history: after catch trials, in which a perceptual decision was withheld because of task instructions, past stimuli evoked repulsive biases on subsequent orientation judgments; after response trials, however, adjustment errors were systematically attracted toward previous decisions.

Other studies have also used a “no response” condition [43] or an “orthogonal” response condition [29] (e.g., reproducing the orthogonal stimulus orientation), revealing persistent attractive biases and excluding any role for the motor response [43] and response item [29]. It is reasonable, however, that in all these experiments a decision about the relevant stimulus attributes was formed and persisted from trial to trial. In fact, stimulus-related decisions are required for relational (orthogonal) judgments and may reflect the default strategy in tasks in which the demand for a response is uncertain [82], but responding is the most likely case [26,43]. Hence, if positive serial dependence arises from persisting internal representations required to perform the task, from trial to trial, it is no surprise that the presence or type of response plays no role.

In line with this idea, here we demonstrated that the same opposite pattern of history biases can be gleaned from discriminative tasks requiring forced-choice responses. The results of our Experiment 4, without adjustment responses, are indeed of critical relevance for two main reasons: (1) They rule out any possibility that our findings may be due to orientation bias confounds in adjustment responses [43,65], and (2) they prove that attractive and repulsive history biases alter directly the sensitivity of our perceptual system and thus the appearance of stimuli. Previous sensory signals may indeed act as an internal reference model against which incoming stimuli are compared [83]. Repulsive biases may contribute to the refinement of trial-by-trial internal references that maximize the system sensitivity during discriminative tasks. Decisional processes, on the other hand, build upon the gathering of sensory evidence over time [84]. When sensory input is weak or scanty (as in our 0% SNR condition), decisional templates may continue to integrate information within the actual decision boundaries, causing a local decrease of perceptual sensitivity.

Forced-choice tasks have been already employed in combination with adjustment responses to probe the perceptual/postperceptual nature of serial dependence, leading however to contrasting results. For example, Fritsche and colleagues [30] found repulsive biases in two-alternative forced-choice (2AFC) tasks when the orientation of 2AFC stimuli was ±20° away from that of a preceding adjustment task. Using a similar procedure but including more values for Δ*S* between adjustment and 2AFC stimuli, Cicchini and colleagues [29] failed to replicate the repulsive bias at ±20° but found positive serial dependence for smaller Δ*S* (e.g., ±5°, 10°). Although our findings may not directly resolve this inconsistency, an interpretation in terms of decision inertia can be proposed. First, decisional processes and readout templates may be different between tasks requiring a qualitative scaling (e.g., orientation adjustment) or comparative/discriminative judgments (e.g., forced-choice tasks), and their degree of overlap and interaction may be reduced—serial dependence may occur only for highly similar stimuli [29]. Second, when fixed and predictable Δ*S* are used (e.g., ±20° [30]), other factors may come into play, including expectations about systematic detectable differences between consecutive stimuli [85–87], which may alter perception, decisional strategies, and sequential effects. It is also worth noting that forced-choice tasks per se are not impermeable to history biases [88] and readout strategies [89] but are more strongly affected by previous decisions/responses than previous stimuli [88]. Hence, the positive serial dependence observed with hybrid tasks [29] may still reflect decisional inertia.

The decreased perceptual sensitivity after decisions in the absence of sensory evidence is in line with a recent work showing that decisional inertia increases the tendency of choice repetition after weak stimuli [53]. This supports the notion that the repulsive/attractive consequences of recent events may depend on the quality of the sensory input [29]; however, it also suggests that two contrasting forces coexist at every instant of perception and originate at different stages of the processing hierarchy. The most compelling evidence of such a hierarchical system emerged from the results of Experiment 5, in which past stimuli and responses were dissociated and both positive and negative aftereffects could be revealed in a single perceptual report. Conditioning errors on past stimuli and responses in this key experiment showed the independent profile of the two sources of serial dependence, replicating the repulsive biases for stimuli observed in Experiment 3 and revealing the attractive impact of decisional traces even after streaks of intervening stimuli.

One may wonder to what extent repulsive biases reflect visual adaptation in tasks with briefly presented and masked stimuli. Whereas the same consideration applies to low-level positive serial dependence, repulsive biases in orientation perception (e.g., the tilt aftereffect [TAE] [41,77,77,90]) and other visual domains [91] are evident even after a few milliseconds of exposure [76,77,92], suggesting that this rapid form of adaptation-induced plasticity may reflect an instantaneous and stereotyped behavior of ensembles of sensory neurons. As a consequence, when adaptation is weak (e.g., after brief stimuli), decisional readout processes prevail. When adaptation is strong (e.g., after prolonged exposure [26]), instead, decisions will be formed by integrating signals from a drastically adapted system, and adaptation prevails. Additional work could further evaluate the involvement of sensory adaptation by presenting sequences of stimuli at different retinal positions: although in our paradigms retinally overlapping stimuli may have promoted repulsive biases when perceptual decisions were not required, it would be interesting to investigate the direction and strength of history biases in the absence of decisional processes and when adaptation is minimized. Here, we showed that the two competing mechanisms can be disentangled by isolating decisional and sensory traces (Experiment 5).

Another possibility is that the observed repulsive bias is not a genuine result of neuronal adaptation but an opposite type of serial dependence that follows response inhibition and stimulus devaluation [93]. Although this is an intriguing possibility that deserves future investigation, the similarity between the repulsive bias in Experiment 3 and the one in Experiment 5 (in which adaptation was facilitated in the absence of backward masking and with short interstimulus intervals) justifies our interpretation of repulsive biases as emerging from low-level adaptation effects. A further intriguing alternative might be that positive and negative history biases result from attentional selection [26,94], which acts as a gating mechanism that enables or blocks the persistence of perceptual traces based on the relevance of sensory events [95]. This seems quite plausible if one assumes that negative biases are the consequence of inhibitory (e.g., below baseline) modulations of early sensory activity induced by the top-down attentional suppression of irrelevant stimuli. However, such suppressive effects of attention are usually observed in response to salient and distracting stimuli [96,97], whereas in our paradigms saliency was equated across all stimuli, and participants were unaware of the relevance/irrelevance of each stimulus until the response stage. Furthermore, in our Experiment 5, we found no evidence that participants were attending to the last, to-be-reported stimulus while ignoring or “suppressing” the preceding one in the sequence (see Methods). Thus, in light of all evidence considered here, decisional inertia still appears the most satisfactory interpretation of positive serial dependence in our results. Nonetheless, we recognize that the involvement or additional role of attention is clearly an interesting question for future work.

To account for the opposite effects of stimuli and decisions, we proposed a Two-process model of serial dependence, combining low-level orientation adaptation and persisting decisional templates in a single hierarchical structure. We modeled low-level adaptation as a repulsive reorganization of a population of orientation-selective neurons [74,98] and the decisional stage as a weighted decoding of low-level information [99–101]. By incorporating a memory trace for readout weights at the decisional stage, we provided a simple model that approximated both low-level adaptation, in the absence of decisional activity, and attractive biases from the decoding stage. The constant reweighting process counteracted and masked the effects of orientation adaptation, leading to the positive serial dependence reported here and in previous work [26,30,63].

The contribution of our work to the perceptual/postperceptual debate about serial dependence depends strictly on the operational definition of perception. If we define perception in terms of stimulus appearance [46] and subjective internal representations, then our results and the Two-process model would certainly classify positive serial dependence as a perceptual phenomenon—it does alter stimulus appearance. What serial dependence is not, according to our perspective, is a process emerging at the earliest stages of sensory encoding. To express this difference in greater details, a parallelism with the PL domain might be useful. PL refers to the improvement in perceptual performance after extensive training, which underlies long-lasting neuronal changes in the brain [102–105]. Although it has long been proposed that such profound changes engage modifications at the level of early sensory neurons (e.g., V1 [106,107]), growing evidence supports the hypothesis that PL reflects changes at higher-level stages [54,108–110]. According to this idea, PL would arise from improved readout connections between higher-level decisional units and lower-level sensory areas [54], without considerable changes at the lower stages. Thus, in PL, recurrent readout processes tune connection weights and lead to measurable improvements in perception over the long term. In a similar fashion, the inertia in updating these trial-by-trial connection weights, postulated by our model, would transitorily lead to changes in stimulus appearance that can be measured over the short term.

The plausibility of our Two-process model is corroborated by extensive evidence of readout processes and integration of sensory information at high-level stages of processing [55,89,99,111] and by the critical role of decisional circuits in recent models of visual perception [54,112]. Furthermore, it is in line with the fact that early visual areas show little to no increase in sustained activity over time [113,114], contrarily to what would be specifically expected from the Gain model [26], although their information content persists longer when engaged in memory and decision-related processes [115–117], whereas task- and decision-related activity reverberates over prolonged delays in higher-level neuronal circuits [57,118,119]. Decisional inertia may therefore reflect the tendency of a specific pattern in decision-making circuits (e.g., a recurrent readout process, perceptual template) to resist changes in its actual state [119]. This inertia may be proportional to the strength (mass) of a decision-making instance. This possibility fits well with the results of our Experiment 6, demonstrating that decisional traces persist more strongly after the sensory/decision circuit has been perturbed with high-contrast stimuli, which may have caused stronger network activity and slower relaxation dynamics [120]. One may therefore speculate that the stronger the intensity (mass) of a past perceptual/decisional episode, the larger its impact on future stimuli (inertia). Similarly, the fact that serial dependence decreased when high-contrast stimuli followed low-contrast stimuli is in agreement with the idea that salient and strong sensory signals can cause resets in decisional circuits, reducing attractive aftereffects [120].

The results of our Experiment 6 are also relevant for the understanding of the interactions between positive and negative biases in visual perception [46]. Whereas stimulus contrast has been previously manipulated in separate experiments [43], with serial dependence occurring only for low-contrast stimuli, the critical interaction between past and present stimulus strength has not yet been tested. Our findings demonstrate that, in the same participant, response-driven serial dependence by low-to-low- and high-to-high-contrast stimuli does not differ. The difference emerged when comparing low-to-high and high-to-low sequential effects, with attractive biases being higher in the latter case. On the one hand, this suggests that strong signals rapidly dissipate persistent traces from previous perceptual/decision-making events. On the other hand, this indicates that although they may promote higher degrees of adaptation, when readout processes still prevail over adaptation, strong signals exert a larger impact on future weak signals.

Hence, our results indicate the existence of two functionally segregated processes of serial dependence that operate at different stages of the processing hierarchy. Attractive biases are not induced by recent stimuli on current responses but are the by-product of chains of decisional processes that are paralleled by repulsive adaptation at a lower stage. The behavior of this hierarchical system generalized also to the domain of ensemble coding [80], demonstrating that the appearance of an ensemble summary is biased toward past decisions more than by the statistical properties of previous stimuli [31].

Although the Two-process model represents a parsimonious and unified account of serial dependence, other alternatives can be formulated. For example, by substituting decisional weights with probability distributions (priors) and low-level population activity with sensory evidence (likelihoods), one can accommodate our hierarchical model into the predictive coding framework [47,121–123]. According to this perspective, higher-level probability distributions built upon past information (e.g., previous stimuli) would be used to predict and test the incoming sensory input. As a consequence, when two consecutive stimuli are similar in some feature domain (e.g., orientation), the product of prior knowledge and current sensory evidence would result in mixed representations. It has been proposed that serial dependence emerges from such a predictive mechanism, educated by environmental priors [47] and by the fact that objects in the real world tend to remain stable over time. This implies that in the absence of a reliable temporal structure, the best prediction of future stimuli is that they will match the current percept [38]. Although this represents a suggestive theoretical proposal, it relies on the strong assumption that the brain systematically expects the reoccurrence of the same stimulus in a random sequence. However, if the past in a random series is used as a reference to build predictions, it seems more plausible that perception, shadowing the human reasoning system, would fall into the “local representativeness” heuristic [124], expecting consistent changes within short sequences of perceptual events [8,125] in order to match an internal model of randomness [126,127]. Along a similar line, it has been suggested that, more than from deep-seated priors for object stability, serial dependence originates from optimal perceptual strategies based on adaptive coding and temporal integration [49]. In this view, human perception would resemble an optimal Bayesian estimator (or “ideal observer” in psychophysical terms) that maximizes performance through a weighted integration of highly similar sensory events over time [49]. Although optimality is a questionable term when referring to a mechanism of sequential integration under a stochastic sequence of sensory events (e.g., independent trials) [128], it is worth noting that the formal aspects of this idea are generally in line with our proposal: irrespectively of whether it produces an optimal or suboptimal behavior, serial dependence arises from an integrative process that carries over information from one trial to the next, biasing stimulus appearance. Here, we show that sequential decisions are fundamental for this integrative process, whereas in their absence, temporal segregation and repulsive biases do prevail.

An alternative explanation of positive serial dependence has been proposed based on working memory biases [30,48]. In our final experiment, we compared the impact of visual working memory content and previous decisions in perceptual errors during size estimation tasks. Although, even at the conceptual level, it is difficult to disentangle between working memory content and persistent decisional processes, our Two-process model would predict that only internal templates that have been used for a decision can affect future perception, independently of the amount of new information in working memory. Confirming this prediction, our results showed that traces of previous decisions produce attractive biases, whereas working memory content had a repulsive effect on perception, in line with similar findings reported before [81,129]. The repulsive bias appeared to be stronger for memorized stimuli in the spatial proximity of the to-be-reported stimulus (Fig 12B).

As a final remark, few neuroimaging studies have investigated serial dependence so far. For example, there has been only a single attempt to address how positive serial dependence is reflected in brain activity [44]. This work, using functional magnetic resonance imaging (fMRI), investigated orientation-selective activity in V1 during a 2AFC task (left/right oblique). The authors found carryover effects from trial to trial that were reflected by activity patterns in the visual cortex. However, as the same authors discussed [44], their paradigm did not allow determination of whether serial dependence reflected stimulus- or decision-related activity, and when incorrect trials were analyzed, they found perceptual biases toward previous (incorrect) choices, more than previous stimuli [44]. Because of the low temporal resolution of fMRI and the fact that it records mainly input to a given area, more than local computations [130], it is plausible that such findings reflect reentrant feedback from higher-level (decisional) structures to V1. A more recent work has found differences in visually evoked electroencephalographic (EEG) responses that resembled serial dependency patterns in numerosity estimation, even in the absence of an explicit task [50]. In scalp data, such differences emerged at a relatively late and restricted time window (at around 200 ms from stimulus onset), whereas no modulations by serial dependence occurred for the early components typically evoked by numerosity magnitude (approximately 100 ms). A subsequent neural decoding analysis revealed earlier signatures of serial dependence (e.g., before 100 ms), but the sign (attractive or repulsive) of this bias was not decoded. Therefore, although the report of serial dependence without an explicit task is intriguing, further neuroimaging and EEG work is required to clearly characterize the sites and dynamics of history biases in the brain.

What is the functional role of the two sources of serial dependence? We argue that both mechanisms serve the adaptive function of maximizing perceptual efficiency over the short- and long-term range. In free viewing conditions, we tend to make relatively rapid and large saccadic eye movements with subsequent fixations likely to land on stimuli with highly dissimilar structures [76]. The rapid and repulsive reorganization of low-level sensory neurons may thus prepare our perceptual system to decorrelate and resolve different spatial structures, enhancing its ability to discriminate local features in a discontinuous perceptual environment. The lingering of decisional traces, on the other hand, may be explained by the integrative nature of decisional processes. Decisions terminate in a choice/response after sensory evidence is accumulated to some threshold level [84]. Decisional processes may therefore have a long integration and decay time that persist from one trial to the next.

Although the persistence of decisional weights can bias perception in the short term, leading to positive serial dependence, it may aid the long-term calibration of optimal decisional weights that fosters PL [54,111,112]. Another intriguing possibility is that the decisional system implicitly counteracts adaptation and compensates for repulsive biases at the lower stage in the attempt to promote visual stability in a top-down fashion.

To conclude, in the present work we provide a unified account of the concatenated history of perceptual and decisional processes. We present a hierarchical model of serial dependence in which two separate and contrasting forces, exerted by adaptation and decisional inertia, interact in shaping visual experience and our reactions to environmental stimuli. Building on recent work [30], our study has strong theoretical and experimental implications in dissociating different sources of history biases in perceptual and psychophysics research.

## Methods

### Ethics statement

The study was approved by the local Ethical Committees of the University of Verona and of the University of Fribourg and carried out in accordance with the Declaration of Helsinki.

### General methods

A total of 110 subjects (79 females, mean age = 23.08 ± 3.93 years)—37 from the University of Verona, Italy, and 73 from the University of Fribourg, Switzerland—participated in this study for monetary payment (7€) or course credits. All participants had normal or corrected-to-normal vision and were naïve as to the purpose of the experiments.

Stimuli were presented on a Multiscan G500 CRT 21” monitor (1,024 × 768 pixels, 100 Hz, Experiments 1, 3, 5, and 6) and on a Philips 202P7 CRT (1,600 × 1,200 pixels, 85 Hz, Experiments 2, 4, and 6) and were generated with a set of custom-made programs written in MATLAB (R2017b) and the Psychophysics Toolbox 3.8 [131], running on Windows XP–based machines. All experiments were performed in dimly lit rooms, and participants sat at a distance of 45 cm (Experiments 1, 3, and 6) or 70 cm from the computer screen, with their head positioned on a chin rest. All stimuli were presented on a gray background (approximately 24 cd/m^2^). Across experiments, the number of trials varied in order to achieve a minimum of 20 data points for each variable of interest, while keeping the overall duration within roughly 1 hour. Participants were instructed to maintain their gaze at the center of the screen for the entire duration of all experiments.

### Experiment 1

An example of a trial sequence in Experiment 1 is illustrated in Fig 1A. Each trial started with a green central fixation spot (0.5°) followed by the presentation of a peripheral Gabor (8.5° to the left or right of fixation). Gabor stimuli had a peak contrast of 50% Michelson, spatial frequency of 0.5 cycles per degree and a Gaussian contrast envelope of 1.5°. After 400 ms, a noise mask appeared at the same location of the Gabor for 400 ms. Masks were white-noise patches of the same size and peak contrast of the Gabor stimuli, smoothed with a symmetric Gaussian low-pass filter (filter size = 1°, SD = 2°). After a blank fixation interval of 500 ms, a response bar was presented at the same location of the Gabor. The response bar was a 0.5°-wide white bar windowed in a Gaussian contrast envelope of 1.5°, with the same peak luminance of the Gabor stimuli. On each trial, the response bar appeared with a random orientation and participants were asked to rotate the bar until it matched the perceived orientation of the Gabor. The response bar was rotated by moving the computer mouse in the upward (clockwise rotation) or downward (counterclockwise rotation) directions, and the final response was confirmed by clicking the mouse left button. After a random interval (400–600 ms), a new trial started.

On each trial, the Gabor was assigned 1 of 18 possible orientations, ranging from 0° (vertical) to 170° in steps of 10°. The series of orientations was pseudorandomly determined in order to keep the relative orientation between consecutive stimuli (Δ*S*, previous orientation minus present orientation) in the range ±50°, in steps of 10°.

At the beginning of the experiment, participants were provided written instructions and performed 20 trials of practice that were not recorded. The experiment consisted of four blocks of 140 trials each, for a total of 560 trials (approximately 30 trials for each orientation tested and approximately 50 trials of each level of Δ*S*), and lasted approximately 1 hour. Stimuli were presented to the right or left of fixation in separate blocks. The average reaction time in all experiments using the same orientation adjustment task (Experiments 1–3, 5, and 6) was 2.23 ± 1.26 seconds.

### Experiment 2

The procedure in Experiment 2 was similar to that in Experiment 1, with the following exceptions. Gabor stimuli were presented at the center of the screen and had higher spatial frequency (1.2 cycles per degree). The orientation of stimuli varied pseudorandomly in the range 0–179° (steps of 1°), and the relative orientation Δ*S* was a continuous variable ranging from −80° to +80° in steps of 1°. There were four blocks of 110 trials for a total of 440 trials.

### Experiment 3

The procedure in Experiment 3 was similar to that in Experiment 1, with the following exceptions. In order to compare our results with previous work showing serial dependence in the absence of prior motor responses [26], we made our paradigm more similar to the one adopted by Fischer and Whitney ([26]; Experiment 2). Gabor stimuli were presented at ±6.5° from fixation for 500 ms, and the duration of the mask was increased to 1,000 ms. In about 40% of the total trials (catch trials), the response bar was replaced by a black disk (1.5° of diameter, 200 ms) explicitly instructing participants not to report the Gabor orientation. The duration of intertrial intervals in catch trials was a running average of individual reaction times during the task. The orientation tested ranged from 0° to 165° in steps of 15°, with Δ*S* of ±60° in steps of 15°. The increase of the step size for orientations and Δ*S* with respect to Experiment 1 allowed us to collect a sufficient number of data points for response trials (approximately 30 for each orientation and approximately 40 for each Δ*S*) and catch trials (approximately 20 for each orientation and approximately 26 for each Δ*S*) while keeping the overall duration of the experiment at about 1 hour.

### Experiment 4

An example of a trial sequence in Experiment 4 is illustrated in Fig 4. The dual task consisted of a 1AFC symmetric task [70], followed by a Yes/No task. Each trial started with a central reminder of the 1AFC task (two oblique “\” and “/” lines presented in red) for 1 second. After the reminder, an oriented Gabor (1 cycle per degree, contrast = 25%, duration = 500 ms) and a subsequent noise mask (contrast = 50%, duration = 500 ms) were shown at the center of the screen. The SNR of the Gabor was determined by keeping a proportion of randomly selected pixels from the Gabor image and filling the rest with uniformly distributed noise. In the 0% SNR condition, the image contained only noise. When the SNR was higher than 0% (25%–75%), the Gabor could have a left oblique orientation (45°) or a right oblique orientation (135°), randomly intermixed and balanced across trials. Participants had to report the left versus right oblique with their left hand, using two dedicated keys in the computer keyboard.

After the 1AFC response (mean reaction times = 559 ± 207 ms) and a blank interval of 500 ms, the reminder for the Yes/No task appeared for 1 second (one vertical and one horizontal line in green). The reminder was followed by a central Gabor (contrast = 50%, SNR = 100%, 500 ms). The orientation of the Gabor deviated from one of the two diagonals (45 or 135°) with eight possible offsets (±2, 5, 15, 40°, with negative values indicating deviations toward the horizontal axis and positive toward the vertical axis). In half of the trials, the reference diagonal for the Yes/No task was the same as the stimulus orientation in the preceding 1AFC task (e.g., 45-to-45° and 135-to-135°, Congruent condition). In the other half, it was the opposite (e.g., 45-to-135° and 135-to-45°, Incongruent condition). In 0% SNR trials, the Congruent/Incongruent conditions were assigned online, depending on the orientation reported by the participant in the 1AFC. Participants had to report whether the Gabor was more tilted toward the vertical or horizontal axis with their right hand, using two dedicated keys in the keyboard. After their response (mean reaction times = 847 ± 184 ms), a random intertrial interval from 500 to 800 ms preceded the next trial.

All participants performed a brief training session (20 trials) before the experiment with task-specific trial-by-trial feedback and were not informed about the absence of a detectable orientation in the 0% SNR condition. There were four blocks of 90 trials each, for a total of 360 trials.

### Experiment 5

To test the predictions made by the two data-generating models of serial dependence, we performed an experiment in which stimuli to be reported were interleaved with sequences of stimuli not requiring a behavioral response. In this experiment we pursued an additional aim to measure the role of expectations in serial dependence comparing response biases after regular (i.e., ABCABC) or random orientation sequences. However, we found no differences between conditions and no explicit learning of the regular sequences, indicating that the employed manipulation of expectation was not effective. Therefore, given the suitability of this paradigm for evaluating the Two-process hypothesis of serial dependence, we used all data from this experiment to test the predictions made by the two data-generating processes.

An example of a trial sequence in Experiment 5 is illustrated in Fig 1B. Gabor stimuli were the same as in Experiment 2 and were presented at the same central location. Eighty percent of trials contained a sequence of six Gabors (200 ms each) with varying orientations (from 0° to 160° in steps of 20°) and a Δ*S* of ± 80°, in steps of 20°. No noise mask was presented after each stimulus, and the phase of the sinusoidal component of each Gabor was changed randomly on each presentation (0–360°, steps of 15°). On the remaining 20% of total trials (catch trials) the sequence ended at a random position before the sixth Gabor (after 1–5 Gabors). These catch trials were used to ensure that participants were paying attention to the entire sequence and, most importantly, to the fifth stimulus before the to-be-reported orientation (fifth versus sixth stimulus error magnitude, *t*_(1,14)_ = 0.28, *p* = 0.78, two-tailed *t* test). After 400 ms from the offset of the last Gabor in the sequence, the response bar appeared at the center of the screen, and participants had to report the orientation only of the most recently seen stimulus. Only trials in which the sequence was completed after six Gabors were analyzed. There were four blocks of 71 trials each, for a total of 284 trials.

### Experiment 6

The procedure, stimuli, orientation steps, and Δ*S* were the same as in the response trials of Experiment 3. The contrast of each Gabor was varied randomly between low (25% Michelson) and high (75% Michelson) on a trial-by-trial basis. The duration of the Gabor was also increased to 1,000 ms, and the following noise mask lasted 500 ms. Reaction times for one subject were not recorded, because of a computer programming error. There were 5 blocks of 80 trials in Experiment 6, for a total of 400 trials.

### Experiment 7

An example of a trial sequence in Experiment 7 is illustrated in Fig 10A. Stimuli were ensembles of 16 light-gray dots of four different sizes. The average diameter of the ensemble was 0.75°, 0.945°, 1.19°, or 1.5° and was randomly assigned on each trial (see [79] for a similar procedure). The SD of the dot diameter within an ensemble was either 0.2° or 0.4°. The dots were arranged in a 4 × 4 matrix (14.1 × 10°), separated from the monitor’s center by 1.5° in the horizontal direction and 2° in the vertical direction. Dots were centered on equally spaced cells of 3.52 × 2.5°, with a random horizontal and vertical jitter ranging from 1 to 16 pixels. The distance of the ensemble’s center from the fixation spot was 11.14°.

Each trial started with a green fixation spot (0.5°) at the center of the screen for 400 ms. Then, the ensemble appeared for 200 ms at one of four possible locations (top left, top right, bottom left, or bottom right). After the ensemble offset, a dark-gray response dot appeared at the center of the screen with a random diameter (ranging from 0.2° to 4°), and participants were asked to adjust the diameter of the response dot to the perceived average size of the ensemble. The adjustment response was performed by moving the computer mouse in the upward (diameter increase) or downward (diameter decrease) directions and then clicking the left button to confirm the response. After a variable interval (500–700 ms), a new trial started. The average reaction time of participants in this experiment was 1.89 ± 0.52 ms. The experiment consisted of 4 blocks of 120 trials, for a total of 480 trials.

### Experiment 8

An example of a trial sequence in Experiment 8 is illustrated in Fig 10B. In two separate versions of this experiment, we used two and four light-gray dots of different sizes. The two dots were presented at ±5° from the monitor’s center. The four dots were arranged at the corners of a central imaginary square (diagonal = 10°). The diameter of each dot was randomly chosen from 50 linearly spaced intervals between 0.5° and 5°.

Each trial started with a central green fixation cross for 500 ms, followed by the presentation of the circles for 200 ms. Participants were instructed to memorize the diameter of each dot until a spatial retro-cue (a black X) appeared in one of the dots’ locations after a fixed delay of 1,000 ms. Participants were then asked to adjust the size of a central response line with random orientation (edges = {0.05°,8°}) to the diameter of the cued circle, using the mouse. After a variable interval (500–800 ms), a new trial started. The average reaction times were 2.19 ± 0.32 seconds with two dots and 1.91 ± 0.34 seconds with four dots. The experiment consisted of 4 blocks of 100 trials, for a total of 400 trials.

### Data analysis

#### Outlier correction

As a first step in the analysis of the data from all experiments, circular errors in the orientation tasks (Experiments 1–3, 5, and 6) were bounded to ±90° with respect to the stimulus orientation, approximating a normal distribution. Trials including and following responses with unrealistically fast (<200 ms) or outlier reaction times (>3.5 SDs) were removed from all experiments (1.91% ± 2.81%). Finally, adjustment errors were demeaned to remove any chronic bias from the performance of each participant (e.g., systematic clockwise or counterclockwise errors in the orientation task or tendencies to report the diameter as larger or smaller in the size estimation tasks), and a Grubbs test [132] was applied to exclude outliers from the data of each participant (α = 0.05). As a consequence of this preprocessing, fewer than 2% of trials were excluded in total, and the participants’ average absolute error was 8.03 ± 1.93° in the orientation tasks and 0.41 ± 0.21° in size estimation tasks. The average proportion of errors in the 1AFC (Experiment 4) was 0.01% ± 0.01%.

#### Multilevel modeling

To avoid the pitfalls of aggregated group-level data [133–135], all analyses (except one in Experiment 6) were performed by means of multilevel, linear, and nonlinear modeling. In Experiments 1, 2, and 7, two competing models were built and compared. In the first model, errors were analyzed as a function of the difference between the orientation/average size of previous and present stimuli (Δ*S*). In the second model, the errors were analyzed as a function of the difference between previous responses and present stimuli (Δ*R*). The two predictors were highly collinear, and it was not advisable to include them in the same model. Therefore, we fitted separate Δ*S* and Δ*R* models to these datasets, and we compared the goodness of their relative fits. In the remaining experiments with noncollinear predictors, the independent variables of interest were included in the same model.

#### Modeling orientation adjustment errors

In the orientation adjustment tasks of Experiment 1 and 2, serial dependence of the errors *y*_*ij*_ was modeled with a nonlinear mixed-effects model of the form:

$$y_{ij}=DoG\left(x_{ij};\;\alpha_{i},w\right)+e_{ij},$$ (1)

with *i* the subject index and *j* the *jth* trial, and *DoG*(*x*_*ij*_; *α*,*w*) is given by:

$$\{\begin{array}{c} DoG\left(x_{ij};\;\alpha_{i},w\right)=x_{ij}\alpha_{i}wce^{-{\left(wx_{ij}\right)}^{2}}\\ \alpha_{i}=\alpha_{0}+\alpha_{1}\;\gamma_{i}+u_{i} \end{array},$$ (2)

where *x*_*ij*_ is the predictor variable (either Δ*S* or Δ*R*) and (*α*_*i*_,*w*) are unknown parameters, with $\alpha_{i}\in R$ the height of the peak and valley of the curve, *w*∈(0,1) the spread of the curve, and $c=\sqrt{2}/e^{-0.5}$ the normalization constant [26]. *α*_*i*_ is formed by a group-level (*α*_0_) component and an individual-level (*α*_1_) component, with *γ*_*i*_ being the subject indicator variable. *u*_*i*_ is the group-level error assumed to be independent and identically distributed as $u_{i}\mathrm{iid}∼N(0,\sigma_{u}^{2});\;e_{ij}\mathrm{iid}∼N(0,\sigma_{e}^{2})$ is the residual model error, independent of *u*_*i*_. The mixed-effects model analysis was performed using the *nlmefit* routine (*MaxIter* = 200, *TolX* = 1e-4, starting parameters: *α* = 2°, *w* = 0.05, *Approximation Type = FO* [Exps 1–2] or *LME* [Exps 3,5–6]) and the *nlparci* function (for confidence intervals on parameter estimates) available in the MATLAB Statistics Toolbox [136].

Before model fitting, the random structure *u*_*i*_ of the model was determined through a model-selection procedure. Because of prior evidence of the effect of Δ*S* and the lack of investigation of the effect of Δ*R*, in the model selection for the random components we used Δ*S* as the independent variable *x*. Model selection was performed by comparing a full model, with both *α* and *w* as random parameters, with a reduced model including only *α*. The estimated random effects for *w* were of small size (<1.0e-03), and the model with only *α* as random effect performed better in both Experiments 1 and 2 (root mean square ([*α*+*w*] − [*α*], Experiment 1 = 0.0007; Experiment 2 = 0.0263). Therefore, in all experiments and for both the estimation of Δ*S* and Δ*R* models, we included only *α* as random effect.

In an effort to determine whether the serial dependence of the errors was associated with the physical stimuli presented in previous trials or with past perceptual decisions (i.e., responses in previous trials), in Experiments 1 and 2 we focused on an exhaustive comparison of the two competing models built with the Δ*S* and Δ*R* predictors. We proved which predictor provided a better fit of the data by means of *rmse*. A nonparametric bootstrap test was used to determine whether there was a significant difference in the *rmse* produced by the two models (*rmse*[Δ*S* − Δ*R*]). We first performed a Monte Carlo statistics by drawing 1,000 bootstrap replicas of two null models (Δ*N*_*1*_, Δ*N*_*2*_), where the Δ*S* and Δ*R* predictors were randomly intermixed across trials: this way, we obtained a surrogate null distribution of *rmse*(Δ*N*_*1*_ − Δ*N*_*2*_) in which differences in *rmse* were not due to the separate ability of the Δ*S* and Δ*R* predictors to fit the data. A corrected *p*-value [137] was then computed as the proportion of *rmse*(Δ*N*_*1*_ − Δ*N*_*2*_) that was higher or lower than the observed *rmse*(Δ*S* − Δ*R*).

In a second step, we used a cross-validation approach to establish the generalizability of the results [138–141]. At each iteration of the process (*n* = 1,000), the cross-validation procedure was based on four sequential steps: (1) The entire dataset was randomly split into train (70% of the data) and test sets (30% of the data). The split was random but took into account the subject stratification (each subject contributed with an equal number of trials in each of the two sets). (2) The Δ*S* and Δ*R* models were separately fitted to the training data. (3) The parameters obtained in step 2 were then employed to generate predictions for the unseen data contained in the test set. (4) The *rmse*(Δ*S* − Δ*R*) was calculated comparing the predicted versus observed outcomes in the test set (see Fig 2D). To evaluate the difference in prediction accuracy between models, we calculated the proportion of iterations in the cross-validation process in which the *rmse*(Δ*S* − Δ*R*) was higher (the Δ*R* model has lower *rmse* and is favored) or lower (the Δ*S* model is favored) than zero. This procedure gave a realistic estimate, in percentage of accuracy, of how reliable is the superiority of the model based on Δ*R*. With a similar aim, in Experiment 5, in which past decisions and stimuli were orthogonal, we compared their relative importance in explaining perceptual errors by means of a dominance analysis [142]. The dominance analysis was reiterated 10,000 times, subsampling from the entire dataset with stratification at the individual level (75%). Since dominance analysis is a well-established method in linear models [142], we used a subset of data in which both predictors (the Δ*S* for nonreported and for reported stimuli) were in the ±40° range (the range in which the DoG approximates a linear function), and we performed the dominance analysis using a linear-mixed model at each iteration. A distribution of the relative dominance (Δ*S*[reported] − Δ*S*[nonreported]) was then obtained with this procedure (see Fig 8D).

The quantification of serial dependence for errors conditioned on both Δ*S* and Δ*R* was based on the estimated parameter *α*, in line with previous studies [26,30,63]. Across all estimated models and for all conditions, the fixed-effect parameter *w* was 0.039 ± 0.018. The statistical significance of the *α* parameter was evaluated by calculating the z-score (the *α* coefficient divided by its standard error) and converting it to a *p*-value by using the standard normal curve [143].

To model serial dependence in experiments with two separate conditions (Experiments 3 and 6), we adapted Eq 1 to the following form:

$$y_{ij}=DoG_{C}\left(x_{ij};C_{ij};\;\alpha_{i},w,k_{i}\right)+e_{ij},$$ (3)

with the multilevel conditional *DoG*_*C*_(*x*_*ij*_;*C*_*ij*_; *α*_*i*_,*w*,*k*_*i*_) given by:

$$\{\begin{array}{c} DoG_{C}\left(x_{ij};C_{ij};\;\alpha_{i},w,k_{i}\right)=x_{ij}\left(\left(\alpha_{i}*C_{ij}\neq 1\right)+\left(k_{i}*C_{ij}=1\right)\right)wce^{-{\left(wx_{ij}\right)}^{2}}\\ \alpha_{i}=\alpha_{0}+\alpha_{1}\;\gamma_{i}+u_{i}\\ k_{i}=k_{0}+k_{1}\;\gamma_{i}+\eta_{i} \end{array},$$ (4)

where *C*_*ij*_ is a dummy variable (0,1) coding for the experimental condition (e.g., *C* = 0 for the Nonreported condition and *C* = 1 for the Reported condition of Experiment 3) and (*α*_*i*_, *k*_*i*_) are the independent height parameters of the curve in the two conditions (e.g., *α*_*i*_ is the height for *C* = 0, and *k*_*i*_ is the height for *C* = 1). As explained above, the height parameter(s) (*α*_*i*_, *k*_*i*_) have a fixed-effect component (*α*_0_, *k*_0_) that indicates group-level performances and an individual-level component (*α*_1_, *k*_1_) that indicates the deviation from the group-level performances. As per above, the variable *γ*_*i*_ is the individual-level indicator and *u*_*i*_ and *η*_*i*_ independent white-noise processes. In a similar vein, we also included an additional parameter *z* to account for variations in the fixed effect (*w*) as a function of *C* (not reported in the formula for the ease of visualization).

Statistical differences between the *α* and *k* parameters (differences between the magnitude of serial dependence across conditions) were assessed by computing a Z-test of the estimated coefficients

$$z=\frac{\alpha -k}{\sqrt{SE\alpha^{2}+SEk^{2}}},$$ (5)

with *SEα* and *SEk* the standard error of the two parameters, and converting it to a *p*-value by using the standard normal curve [143].

#### Orientation bias confound

Orientation judgments are subject to systematic biases in the form of repulsive shifts away from the cardinal and oblique axes [73,144] (see Fig 2E). As warned by previous work, such biases, which depend exclusively on the present stimulus orientation, may generate spurious serial dependency effects that falsely mimic attractive biases on Δ*R* [43,65]. As a consequence, serial dependence has been canonically measured only for previous stimuli (but see [28]).

To evaluate the degree of artefactual serial dependence in our first two experiments, where previous responses were used as predictors of perceptual errors, we ran a Monte Carlo bootstrap procedure in which the model with Δ*R* was estimated 1,000 times, shuffling the temporal order of the orientations (along with their associated responses) across trials. This way, any temporal relationship (i.e., true serial dependence) between previous responses and actual stimuli was removed, and only spurious effects caused by the orientation bias alone remained. In addition, we also quantified spurious serial dependence for one response in the future, an approach that has been used as a control analysis in previous studies [37] (see Fig 2F).

In Experiment 1, we found significant spurious serial dependence after shuffling (α = 0.64 ± 0.37°, *p* < 0.05) and for future trials (α = 1.69°, *p* < 0.05), with a difference between the two (*p*[shuffling higher than future trial] < 0.01). In Experiment 2, spurious serial dependence was significant both after shuffling (α = 2.25 ± 0.33°, *p* < 0.05) and for future trials (α = 2.66°, *p* < 0.05) but without a difference between the two (*p* > 0.05). Since the goal of these experiments was to predict serial dependence based on previous stimuli and responses, the presence of artefactual dependencies for previous responses represented a strong confound. Therefore, to address this issue, we used a stringent approach to remove orientation biases from adjustment responses.

In previous studies, similar issues have been addressed by removing biases after fitting behavioral errors with polynomial functions of different degrees (e.g., fourth-degree [64] or even tenth-degree [43] polynomials). This procedure of residualization entails the estimation of a best-fitting function capturing the systematic shape of response biases. After removing the errors predicted by the estimated function, the “residual” observations are used as bias-free behavioral responses [43,64]. Since orientation biases are known to take a sinusoidal form over the orientation range [43], in the present work we used a sinusoidal fit—a sum of three sinusoids—in the residualization procedure, which performed better than a fourth-degree polynomial fit in both experiments (root mean square of residuals after polynomial versus sinusoidal fit in Experiment 1 = 2.69 versus 1.43, and in Experiment 2 = 4.80 versus 2.19, all *p* < 0.01; all fits were performed using the ‘*fit*’ function in MATLAB, with ‘*Normalize*’ = ‘*on*’, downweighting outliers of ±3 SD from the error mean to 0.1). As the representative case of Experiment 2 shows (see Fig 2E and 2F), this method efficiently removed the systematic biases from orientation responses, wiping out any spurious serial dependence on Δ*R*. This was confirmed by the fact that, after this correction, no significant serial dependence emerged from the shuffling procedure (Experiment 1, α = 0.12 ± 1.98°; Experiment 2, α = 0.01 ± 0.99°, all *p* > 0.05) with no difference between spurious effects in shuffling and future trials (Experiment 1, α = 0.81°; Experiment 2, α = 0.37°, all *p*[shuffling higher than future trial] > 0.05), whereas the observed true serial dependence (Experiment 1, α = 1.71°; Experiment 2, α = 1.45°; see Results) was significantly higher than both (all *p* > 0.05). Residualization was therefore used successfully in these first two experiments. In the remaining experiments with orientation adjustment responses, we used exclusively Δ*S* as the predictor of serial dependence.

#### Modeling *d*’ in forced-choice responses

In Experiment 4, data from the Vertical/Horizontal task were analyzed as a function of the deviation of orientations from the diagonal (±2, 5, 15, 40°) and of the SNR and Congruent/Incongruent conditions in the preceding 1AFC. Responses from the Vertical/Horizontal task were converted into hits and false alarms by classifying them as Yes/No vertical responses [70] (therefore, we refer to this task as a Yes/No task throughout the manuscript). The *d*’ index was then calculated from the hit and false alarm rates using a standard correction for extremes rates (e.g., 0 and 1) [145]. *d*’ values for each level of the Deviation and SNR variables were then submitted to a linear mixed-effects model of the form:

$$\begin{array}{c} y_{ij}=\beta_{0i}+\beta_{1}x_{ij}+\beta_{2}\Gamma_{ij}+\beta_{3}(x_{ij}•\Gamma_{ij})+e_{ij}\\ \beta_{0i}=\beta_{00}+\beta_{01}\;\gamma_{i}+u_{i} \end{array}$$ (6)

where *β*_0*i*_ is the intercept, *β*_1_, *β*_2_ are the slopes associated with the orientation deviation off diagonal (Deviation, *x*_*ij*_) and SNR (*Γ*_*ij*_), and *β*_3_ is the parameter associated with the interaction Deviation × SNR. The intercept *β*_0*i*_ had a multilevel structure, with *β*_00_ representing the group-level fixed effect; *β*_01_ representing the individual-level parameter coded in the variable *γ*_*i*_; and *u*_*i*_ representing an associated white-noise parameter $u_{i}\mathrm{iid}∼N(0,\sigma_{u}^{2})$. In the model, $e_{ij}\mathrm{iid}∼N(0,\sigma_{e}^{2})$ denotes the residual error.

Data from the Incongruent and Congruent conditions were analyzed with two separate models. A first analysis was performed on the criterion index (*c*), revealing no differences across SNR in the two conditions (all *p* > 0.05) but a slight bias to report more often “vertical” in both the Incongruent (*β*_0_ = −0.63, *p* < 0.001) and Congruent conditions (*β*_0_ = −0.74, *p* < 0.001) that decreased as the deviation from diagonal increased (*β*_1_ [Incongruent] = 0.017, *p* < 0.001; *β*_1_ [Congruent] = 0.020, *p* < 0.001).

To evaluate changes in *d*’ for the Congruent condition, a baseline *d*’ was estimated as the average *d*’ across all SNR and Deviation levels in the Incongruent condition. The baseline was then subtracted from an overall measure summarizing the *d*’ for each SNR level of the Congruent condition (collapsing for the Deviation factor, see Fig 5C).

In the analysis of the raw proportions (see Fig 5D), proportions of vertical responses in the two conditions of interest (Congruent-SNR = 0% and Congruent-SNR = 75%) were subtracted from the average proportions in the Incongruent condition (Incongruent minus Congruent) and fitted to a multilevel conditional DoG model (Eqs 3 and 4).

#### Modeling errors in ensemble coding

In the ensemble coding task of Experiment 7, the error *y*_*ij*_ was modeled with a linear mixed-effects model of the form:

$$\begin{array}{c} y_{ij}=\beta_{0i}+\beta_{1}x_{ij}+e_{ij},\\ \beta_{0i}=\beta_{00}+\beta_{01}\;\gamma_{i}+u_{i}, \end{array}$$ (7)

where *β*_1_ *and β*_0*i*_ are the slope and intercept and *x*_*ij*_ the independent variable. The intercept *β*_0*i*_ had a multilevel structure as in Eq 6. The Δ*S* and Δ*R* predictors were included as the independent variable *x* in two separate models.

#### Modeling errors in size estimation

In the size estimation tasks with two and four dots, we used the same linear-mixed modeling approach as in Experiment 7 with the following exceptions. When there were only two circles, the error *y*_*ij*_ (the difference between the reported diameter and the diameter of the target [cued] circle) was modeled as a function of Δ*C* (the size of the competitor stimulus) and Δ*R*. When there were four circles, the error *y*_*ij*_ was modeled as a function of ΔC(1), ΔC(2), ΔC(3) (with ΔC(3) = the furthest competitor), and Δ*R*.

#### Ensemble coding and size estimation confounds

Size adjustment tasks are also subject to systematic confounds. Distortions due to central tendency, guessing, or motor strategies may indeed affect behavioral reports and falsely mimic attractive or repulsive history effects. Therefore, confounds in experiments involving size estimation (Experiments 7 and 8) were screened and removed by operating the following bias-residualization procedure: (1) We fitted a linear model to subject’s error (reported size minus actual size) as a function of the size (Experiment 8) or average size (Experiment 7) of stimuli; (2) subsequently, we subtracted the estimated linear model from the distribution of errors produced by the subjects. As a result, we obtained size estimation errors residualized by any systematic bias [43,64].

Before residualization, 76.6% ± 20.8% of the participants (across the experiments with size estimation) showed some form of systematic bias: 20% ± 8.9% showed central tendency biases (slope < 1), whereas 56.6% ± 23% showed systematic errors toward the edges (slope > 1). After residualization, the estimation of the average size was accurately calibrated (slope not significantly different from unity, slope = 0.99 ± 0.002, CI 0.97–1.02).

In addition, as a further control to exclude any at-chance performance across participants, we assessed overall accuracy by comparing the average absolute error of each participant to an empirical measure of chance performance [146]. The measure of chance performance was obtained by pairing 10^4^ times a given response with the correct response on a randomly selected trial. All participants in all experiments involving size estimation showed a level of precision that was significantly higher than our empirical measure of chance (all *p* < 0.001).

#### Data-generating processes of serial dependence

In the following, we describe and evaluate the predictions of two opposite explanatory processes for serial dependence: the Gain model and the Two-process model. Both data-generating processes were composed by a three-layer architecture including (1) a labeled line-like population of orientation-selective units [98], (2) one decisional unit, and (3) one response stage. Low-level units were 180 circular normal distributions centered on orientations from 0° to 179° in steps of 1° (see Fig 6A[i]). The response of each unit, *r*_*θ*_ to an input orientation *θ* was defined as:

$$r_{\theta }=\alpha e^{\left(\beta \left(\cos\left(\theta -\theta_{0}\right)-1\right)\right)},$$ (8)

where *α* is the amplitude or peak response, *β* regulates the width of the tuning function, and *θ*_0_ is the unit preferred orientation [74,98].

In the absence of any stimulus and decision history, the default parameter values in both models were *α* = 1 and *β* = 4.68 (corresponding to a full width at half height of 28.2° [26]). The collective response of low-level units defined a vector—the population profile—in which each channel signaled its preferred orientation with a value proportional to its response (the closer the input to the preferred orientation, the larger the response, see Fig 6A[ii]). The decisional stage was modeled following previous work and assuming that a decision unit reads out and integrates the population profile [99–101] by computing a weighted circular mean [54,98] (see Fig 6A[iii]), with starting uniform weights of 0.0056 (integrating to 1 over the 180 orientations). The decoded orientation was then relayed to the response unit for the final outcome.

The Gain and Two-process models differed in their respective operating mechanisms when considering the temporal evolution of a series of stimuli and their associated responses. In the Gain model, a gain change was applied to the tuning function of low-level units after the presentation of each stimulus.

The gain change was modeled by substituting *α* in Eq 8 with *G*, where

$$G=1+\alpha_{g}e^{\left(\beta \left(\cos\left(\theta_{n-1}-\theta_{0}\right)-1\right)\right)},$$ (9)

with *β* = 4.68, *θ*_*n*−1_ the orientation just presented, and *α*_*g*_ (the gain factor) as a free parameter. This altered the sensitivity of low-level units (modifying the starting amplitude *α* in Eq 8) and increased the responsivity of those centered onto recently seen orientations (see Fig 6B), resulting in the attractive bias of serial dependence [26]. Decisional weights remained uniforms in the Gain model and had no effect on stimulus decoding.

In the Two-process model of serial dependence, low-level adaptation was regulated by a similar change in the sensitivity and starting amplitude of low-level units, but with inverted sign:

$$A=1-\alpha_{g}e^{\left(\beta \left(\cos\left(\theta_{n-1}-\theta_{0}\right)-1\right)\right)},$$ (10)

thus inhibiting the response of units tuned to recently seen orientations. This caused the repulsive shifts in the responses of low-level orientation channels observed after adaptation [98] (see Fig 6C). The decoding (and report) of stimulus orientation altered the readout weights of the decision unit according to

$$w_{j}=\frac{\left(c+\alpha_{w}e^{\left(\beta_{w}\left(\cos\left(X-R_{j}\right)-1\right)\right)}\right)}{\sum \left(c+\alpha_{w}e^{\left(\beta_{w}\left(\cos\left(X-R_{j}\right)-1\right)\right)}\right)},$$ (11)

where the reweighting *w* in trial *j* is a function of *α*_*w*_ and *β*_*w*_, two free parameters reflecting the peak and width of a circular distribution over the whole orientation range *X*—i.e., the tuning function of the decisional template—and the decoded/reported orientation *R*_*j*_, which sets the center of the new weights distribution (see Fig 6C, green distribution). The constant *c* constitutes a small baseline offset (*c* = 0.1) to avoid zero weights. The denominator is a normalization factor ensuring that the weight distribution integrates to 1.

The tenet of this model was the persistence of past decisional templates after perceptual decisions and behavioral responses *R*_*j*_. The evolution of decisional weights from trial to trial was modeled with the update equation:

$$W_{j+1}=\{\begin{array}{l} \left(W*W_{decay}\right)+w_{j}\;if\;\exists \;R_{j}\\ W\;otherwise \end{array},$$ (12)

where *W*_*j*+1_ is the new set of weights affecting the readout of future stimuli; *W*_*decay*_ is a free forgetting parameter, which determines how fast the history of weights *W* relaxes back to uniform; and *w*_*j*_ is the last reweighted distribution of Eq 11. Hence, in the Two-process model, previous perceptual decisions acted as attractors for reading-out new sensory information.

The optimization of the free parameters was based on the following assumptions: (1) The variation of the parameter *α*_*g*_ in Eq 9 (Gain model) or 10 (Two-process model) should affect the sensitivity of early sensory units (orientation-selective channels) and modulate the magnitude of positive serial dependence or adaptation independently of computations at later stages. (2) The decisional template and its tuning parameters (the amplitude *α*_*w*_ and width *β*_*w*_) are peculiarities of the Two-process model and determine how positive serial dependence emerges from decision inertia. The optimal *α*_*w*_ and *β*_*w*_ should allow decisional templates to counteract adaptation, in the event of repulsive biases at the lower stage. (3) Trial-by-trial variations in the shape of the decisional template (and the resulting model response) are partly due to the persistence of more remote templates (those from trial *n*-2, *n*-3, etc.) whose decay (the forgetting factor *W*_*decay*_) should be chosen in order to approximate the history of behavioral errors in real data.

We therefore optimized the two data-generating processes according to three steps: (1) We first evaluated how varying *α*_*g*_ in Eqs 9 and 10 affected the magnitude of serial dependence (Gain model, Eq 9) or adaptation (Eq 10) in the absence of reweighting processes at the decisional stage. A range of values for *α*_*g*_ (0.05–0.95, steps = 50) was tested in two separate simulations (*n* = 10,000 for both Eqs 9 and 10), and a DoG function was fitted to the simulated errors at each iteration, thus obtaining a relationship between values of *α*_*g*_ and the magnitude of positive—or negative—history biases (see Fig 6D). (2) We then optimized the tuning function of the decisional template for the Two-process model by varying *α*_*w*_ (0–1, steps = 100) and *β*_*w*_ (1–50, steps = 100) independently while minimizing the least-square difference between simulated errors on Δ*S* (*n* = 10,000) and the average profile of positive serial dependence observed after response trials in our first three experiments (α[Δ*S*] = approximately 1.35°, w(Δ*S*) = 0.05°). In this step, we assumed the presence of a small degree of adaptation, which in spite of the short stimulus duration and noisy masks, still promoted repulsive biases at the lower level [30,92]. Hence, we implemented Eq 10 with a gain factor *α*_*g*_ = 0.14 (chosen from step 1) leading to a baseline negative aftereffect of approximately 80°, in line with the magnitude of repulsive biases observed in nonresponse trials of our Experiment 3. The estimated optimal parameters for the tuning function of the decisional template were *α*_*w*_ = 0.08 and *β*_*w*_ = 24.5 (corresponding to an SD of approximately 11°, see Fig 6E). (3) In a final step, we varied *W*_*decay*_ (0.01–1, steps = 100) holding the decisional template obtained in step 2 and the baseline adaptation (*α*_*g*_ = 0.14) in the attempt to minimize the least-square difference between the true single-trial errors made by participants in Experiments 1 and 2 and those predicted by the model. The optimal value for *W*_*decay*_ was 0.19 (see Fig 6F).

With the optimal parameters’ setting, we then evaluated the predictions of the two models in two separate contexts. First, we generated artificial subjects responding to the trial sequences of Experiments 1 and 2 (in a combined dataset) according to either the Two-process or the Gain model. A noise component *ω* was added to the model response *R*_*j*_, with $\omega \mathrm{iid}∼N(0,\sigma_{\omega }^{2})$ and *σ*_*ω*_ = 10°, leading to an average absolute error of approximately 8°, close to the one observed across experiments (8.03°). This data-generating process was reiterated 1,000 times, and the errors of artificial subjects were conditioned on both Δ*S* and Δ*R* (as in Experiments 1 and 2). The difference in the *rmse* of the Δ*S* and Δ*R* fits (Δ*S* − Δ*R*) was collected at each iteration, allowing a direct comparison between the predictions of the Two-process/Gain models and the results of our Experiments 1 and 2 (see Fig 7A).

In a second stage, we tested the predictions of the two data-generating processes in the context of Experiment 5, in which previous decisions and past stimuli were orthogonalized by the specific paradigm. The parameter settings were the same as in the simulation of Experiments 1 and 2 except for a small increase in the degree of expected adaptation, justified by the absence of noisy masks and short interstimulus intervals in Experiment 5. Thus, we imposed *α*_*g*_ = 0.33 in Eq 10, which, from the results of our model optimization (step 1), corresponded to a magnitude of repulsive biases of approximately 1.80°, in line with a constant reported in previous work [92]. The same gain factor *α*_*g*_ = 0.33 led to a positive serial dependence of about 1.20°, approximating the overall amplitude observed in our study. We therefore chose the same *α*_*g*_ for the Gain model and for adaptation in the Two-process model (see Fig 6D). To evaluate the predictions of the two models, we performed a simulation with 10,000 trials having the same structure as in Experiment 5, with sequences of five irrelevant stimuli preceding the to-be-reported orientation.

## Abbreviations

1AFC: one-alternative forced-choice
2AFC: two-alternative forced-choice
AIC: Akaike information criterion
Δ*C*: competitor(s) relative size
DoG: derivative of Gaussian function
Δ*R*: delta response
Δ*S*: delta stimulus
EEG: electroencephalographic
fdr: false discover rate
fMRI: functional magnetic resonance imaging
PL: perceptual learning
rmse: root-mean-square error
SNR: signal-to-noise ratio
TAE: tilt aftereffect
TLRT: theoretical likelihood ratio test
